# Engineered Carbon Dots from a Traditional Herb Pair Orchestrate Concurrent Antioxidant and AP‐1‐Mediated Inflammation to Attenuate Renal Ischemia‐Reperfusion Injury

**DOI:** 10.1002/advs.75596

**Published:** 2026-05-12

**Authors:** Bixiao Liu, Fuying Zhu, Zhuqing Wang, Jiawen Chen, Xiaomiao Cui, Congzhong Yang, Yao Peng, Dengyuan Feng, Li Lu, Hui Wei, Xiaozhi Zhao

**Affiliations:** ^1^ Department of Andrology Nanjing Drum Tower Hospital the Affiliated Hospital of Nanjing University Medical School Nanjing Jiangsu China; ^2^ Department of Biomedical Engineering College of Engineering and Applied Sciences Nanjing National Laboratory of Microstructures Jiangsu Key Laboratory of Artificial Functional Materials Nanjing University Nanjing Jiangsu China

**Keywords:** acute kidney injuries, astragalus membranaceus and angelica sinensis, carbon dots, immune cell infiltration, reactive oxygen species

## Abstract

Renal ischemia‐reperfusion (I/R) injury, a primary cause of acute kidney injury (AKI), is driven by a self‐amplifying loop of oxidative burst and immune cell infiltration. In this work, the classical herb pair *Astragalus membranaceus* (AM) and *Angelica sinensis* (AS) is employed as a composite precursor to synthesize nitrogen‐rich carbon‐dot nanozymes (AM‐AS@CDs) via a one‐step hydrothermal method. AM‑AS@CDs exhibit superoxide dismutase (SOD)‐mimetic activity superior to that of CDs derived from single herbs. In vitro, AM‐AS@CDs effectively alleviate oxidative stress‐induced cellular damage and significantly inhibit apoptosis. In vivo, AM‐AS@CDs markedly attenuate I/R‐induced AKI and reduce immune cell infiltration in renal tissues. Transcriptomic analyses reveal that AM‐AS@CDs downregulate the expression and phosphorylation of Fosl1 and c‐Jun. Consequently, the AP‐1‐chemokine signaling axis is disrupted, which reduces immune cell recruitment. Furthermore, AM‐AS@CDs restore redox homeostasis by upregulating antioxidant enzymes such as SOD, glutathione peroxidase 4 (GPX4), and catalase (CAT). They also attenuate the excessive activation of the Nrf2/HO‐1 pathway. Overall, this precursor‐formulation strategy enables precise modulation of heteroatom doping and surface chemistry in CDs. AM‐AS@CDs effectively interrupt the oxidative‐inflammatory positive feedback loop through dual mechanisms. These findings provide both theoretical and material foundations for the development of natural product‐based nanozymes for I/R‐related diseases.

## Graphical abstract

This study integrates two complementary traditional Chinese medicine components as precursors to synthesize bioactive AM‐AS@CDs. Compared with single‐component CDs, these dual‐component CDs exhibit enhanced antioxidant capacity and superior renoprotective effects. Mechanistically, AM‐AS@CDs suppress AP‐1 signaling by downregulating c‐Jun and Fosl1, thereby reducing inflammation in acute kidney injury. These findings highlight AM‐AS@CDs as a promising nanozyme platform for renal protection.

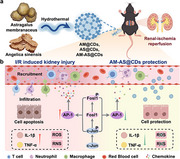

## 1 Introduction

Acute kidney injury (AKI) represents a critical global health burden with high morbidity and mortality, frequently progressing to chronic inflammation and irreversible renal fibrosis [1]. Current estimates indicate that AKI affects more than 13 million individuals annually worldwide and contributes to approximately 1.7 million deaths [2]. Epidemiological data show an AKI incidence of 13%–18% among hospitalized patients, rising to 30%–50% in the intensive care unit (ICU) setting [3]. Among its multifactorial etiologies, renal ischemia‐reperfusion (I/R) injury is the predominant trigger [4, 5]. The mechanism of I/R‐AKI is orchestrated by a complex interplay of molecular cascades, wherein oxidative stress emerges as a pivotal driver [6, 7]. During ischemia, hypoxia suppresses oxidative phosphorylation and leads to the concomitant downregulation of the antioxidant enzyme superoxide dismutase (SOD) [8, 9]. Upon reperfusion, sudden reoxygenation induces an excessive burst of reactive oxygen species (ROS) [10]. The excessive ROS overwhelm endogenous SOD‐mediated detoxification and amplifies oxidative damage [11]. ROS overproduction subsequently triggers maladaptive cellular responses, such as programmed cell death and pro‐inflammatory signaling cascades [12, 13]. Critically, this oxidative burst acts as a primary instigator for the subsequent inflammatory cascade, which plays a critical role in ischemic AKI progression.

During renal I/R injury, tubular epithelial cells represent the primary target, rapidly releasing damage‐associated molecular patterns (DAMPs) as well as a discrete repertoire of chemokines upon reperfusion [14, 15]. This “danger‐chemokine” cascade drives the recruitment of circulating polymorphonuclear leukocytes, macrophages, and lymphocytes into the renal interstitium. Importantly, the extent of infiltration is closely correlated with functional deterioration in AKI patients [16, 17, 18]. Neutrophils are the first to be recruited under the guidance of a CXCL1 gradient. They amplify tissue injury through ROS production and neutrophil extracellular traps (NETs). They also upregulate CXCL1, forming a self‐amplifying positive feedback loop [16]. Subsequently, the CCL2‐CCR2 axis mediates monocyte/macrophage infiltration. Activated M1 macrophages then secrete IL‐1β and TNF‐α, which further propagate the inflammatory cascade [17]. At later stages, lymphocytes infiltrate the kidneys under the guidance of chemokines such as CCL20 and differentiate into various subtypes [18]. This not only exacerbates the tissue damage during the acute phase but also promotes the transformation of AKI into chronic kidney disease (CKD) through sustained T cell responses in the post‐AKI kidneys. Consequently, blocking key chemokine/cytokine axes can effectively attenuate immune cell infiltration [19]. This strategy also mitigates AKI severity and improves long‐term renal outcomes [20].

Oxidative stress serves as a central driver that initiates and amplifies inflammation in I/R‐AKI. Given the absence of specific therapies beyond renal replacement, there is an urgent need for novel interventions capable of disrupting the oxidative‐inflammatory positive feedback loop. Under this premise, nanomaterials with intrinsic antioxidant enzyme‐mimicking properties present a promising therapeutic avenue [21, 22, 23]. Carbon dots (CDs), a class of carbon‐based nanomaterials, have garnered significant attention in biomedicine due to their small size, good biocompatibility, abundant surface functional groups, and tailorable enzyme‐mimicking catalytic activities [24, 25, 26]. Notably, certain CDs can mimic the functions of SOD or catalase (CAT), efficiently scavenging ROS, which makes them potential therapeutic agents targeting the root cause of I/R injury [27, 28, 29, 30]. Recent studies have documented that CDs derived from Chinese medicinal herbs exhibit superior bioactivity compared to their parent herbs [31, 32]. However, a significant limitation persists in current strategies. Most research to date has relied on single medicinal herbs as precursors for CD synthesis. This approach may not fully capitalize on the holistic therapeutic principles of Traditional Chinese Medicine (TCM), which often employs herb combinations to achieve synergistic effects [33, 34]. A critical and unexplored question remains: How can the pharmacological resources of multiple complementary natural medicines be rationally integrated at nanoscale to create composite CDs? Such a design could potentially engineer synergistic interactions directly into the nanostructure, thereby surpassing the catalytic activity and therapeutic efficacy achievable with single‐component herb‐derived CDs.

This study aims to address this gap by developing composite CDs from a binary herb system. *Astragalus membranaceus* (AM) and *Angelica sinensis* (AS) form a classic herb pair (Qi‐Gui herb pair) in TCM [33], which possesses multiple pharmacological activities, including immunomodulation, anti‐inflammation, and antioxidation. They can ameliorate the ischemic‐hypoxic microenvironment and mitigate oxidative stress and inflammatory injury by regulating signaling pathways such as PI3K/Akt, Nrf2/HO‐1, and NF‐κB [34, 35]. Importantly, beyond their pharmacological synergy, AM and AS also provide complementary chemical compositions for nanomaterial construction. AM is rich in polysaccharides and saponins [36]. In contrast, AS contains abundant phenolic acids and small aromatic molecules [37]. This difference leads to diverse carbon precursors. It enables the formation of heterogeneous carbon frameworks, including sugar‐derived and aromatic‐derived structures. Such structural diversity is beneficial for generating abundant surface functional groups [38]. It may also promote heteroatom doping during carbonization [39]. These features are closely associated with the formation of catalytically active sites [40, 41]. They are also critical for enzyme‐mimicking activities, especially ROS scavenging and SOD/CAT‐like performance [42]. We hypothesized that using the AM‐AS herb pair as a mixed precursor could produce CDs (AM‐AS@CDs) with enhanced bioactivity. These nanomaterials are expected to show improved nanozyme‐like catalytic performance and therapeutic efficacy against I/R‐AKI.

**FIGURE 1:**
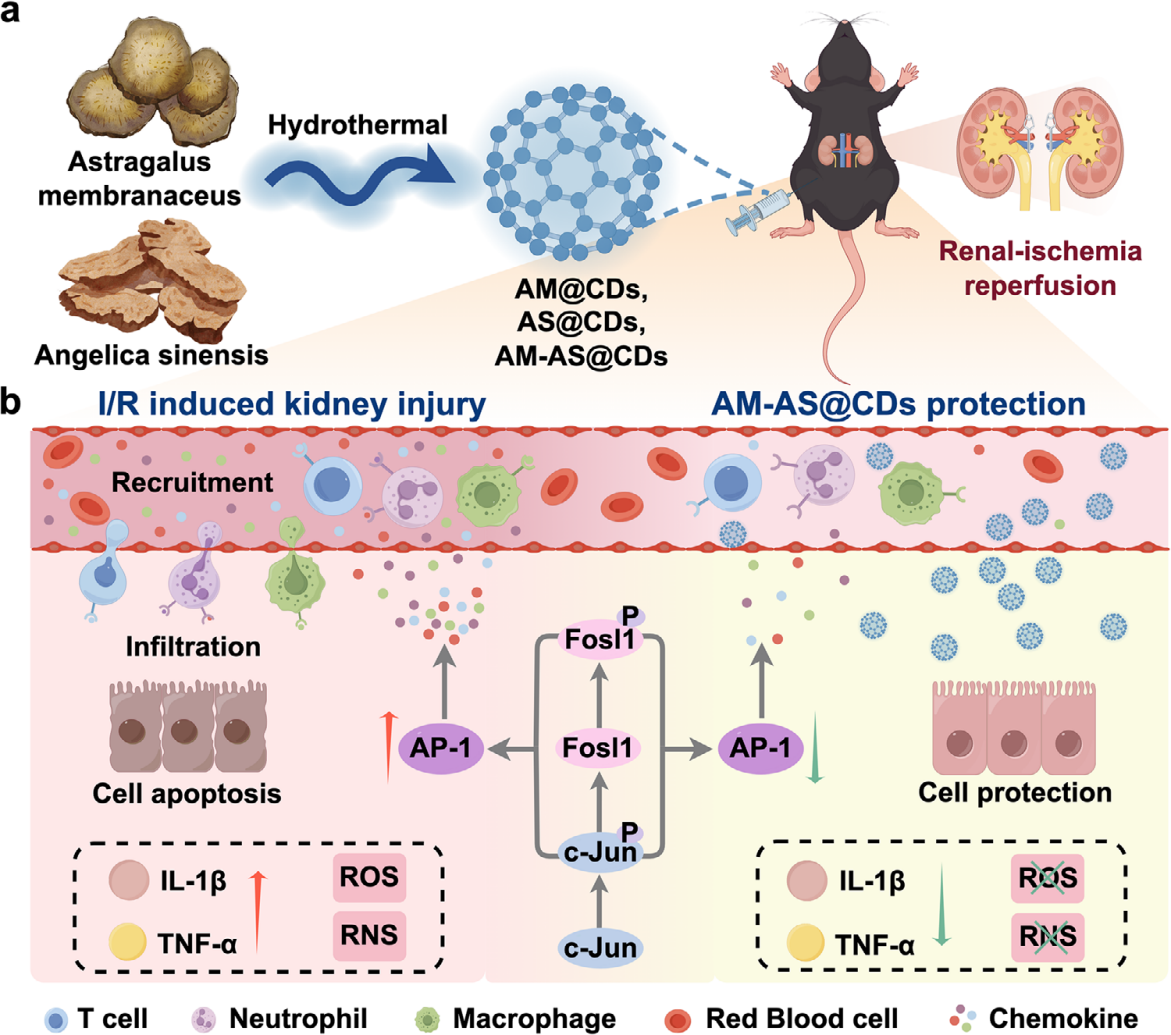
Schematic illustration of the synthesis of AM‐AS@CDs and their mechanism in alleviating renal I/R injury. (a) Synthesis of AM@CDs, AS@CDs, and AM‐AS@CDs via a hydrothermal method. (b) During the treatment of renal I/R injury, AM‐AS@CDs effectively scavenge excessive ROS and inhibit AP‐1‐mediated immune cell infiltration and renal inflammatory damage by downregulating the expression and the activation of c‐Jun and Fosl1.

This study innovatively employs a mixed precursor of AM and AS to synthesize AM‐AS@CDs, systematically comparing them with CDs prepared from the single herbs alone (Figure 1). The results demonstrate that AM‐AS@CDs exhibit superior SOD‐like activity compared to single‐herb‐derived CDs. This indicates that the integration strategy based on TCM compatibility enables complementary and synergistic amplification of pharmacological components within the nanostructure, resulting in enhanced antioxidant and anti‐inflammatory functions. In vitro assays demonstrated that AM‐AS@CDs efficiently scavenge ROS and attenuate apoptosis. Flow cytometric profiling and RNA‐seq analyses further revealed that AM‐AS@CDs markedly curb immune cell infiltration into the kidney in murine I/R injury models. Mechanistically, these CDs disrupt the oxidative‐inflammatory positive feedback loop via two coordinated pathways. First, they attenuate ROS overproduction while preserving endogenous antioxidant gene expression to relieve oxidative stress. Second, they down‐regulate the transcription factors Fosl1 and c‐Jun to interrupt the AP‐1‐chemokine axis, thereby curbing inflammatory cell recruitment. Consequently, the CDs achieve tubule‐specific protection and reprogram the renal immune microenvironment. With high biosafety, exceptional bioactivity, and a facile synthetic protocol, these biomass‐derived CDs offer a promising therapeutic strategy for I/R‐induced AKI.

## 2 Results and Discussion

### 2.1 Design, Synthesis, and Structural Characterization of CDs

We prepared AM‐derived CDs (AM@CDs), AS‐derived CDs (AS@CDs), and AM‐AS composite CDs (AM‐AS@CDs) using AM and AS as herbal precursors via a hydrothermal method. As illustrated in Figure S1, powders of AM, AS, and their mixture were separately suspended in ultrapure water. Each solution was then transferred into a Teflon‐lined autoclave and subjected to a hydrothermal reaction at elevated temperature for a defined period. After cooling to room temperature, the resulting brown‐yellow solutions were filtered and freeze‐dried to obtain the corresponding CDs.

**FIGURE 2:**
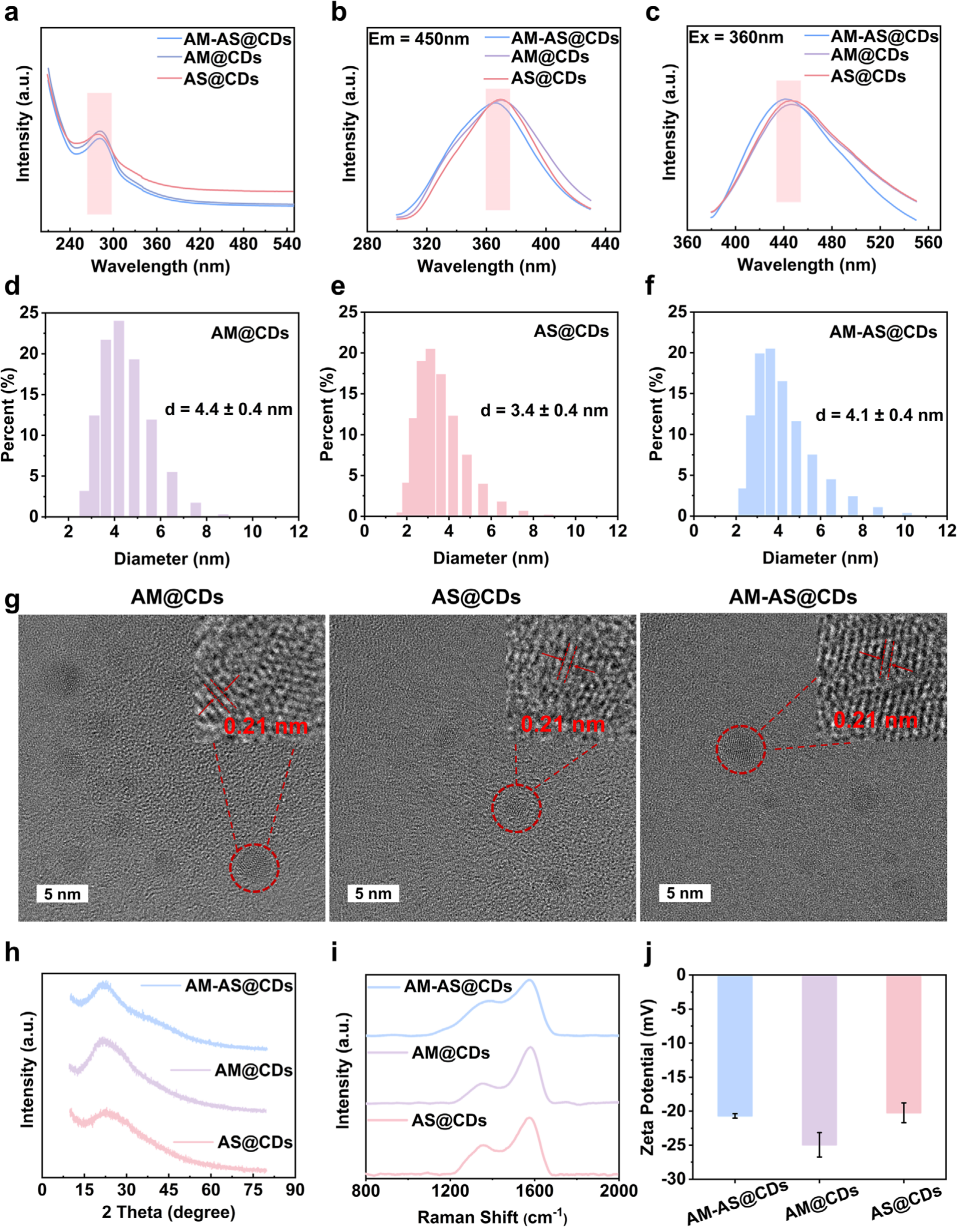
Optical properties and structural characterization of AM@CDs, AS@CDs, and AM‐AS@CDs. (a) UV–vis absorption spectra and (b), (c) fluorescence excitation/emission spectra of the three CDs. (d–f) Hydrodynamic size distributions of (d) AM@CDs, (e) AS@CDs, and (f) AM‐AS@CDs. (g) High‐resolution TEM (HRTEM) image of AM@CDs, AS@CDs, and AM‐AS@CDs (insets: corresponding particle size distributions). (h) XRD patterns, (i) Raman spectra, and (j) Zeta potentials of the three CDs.

As shown in Figure 2a, AM@CDs, AS@CDs, and AM‐AS@CDs each exhibit a distinct absorption peak around 280 nm. This peak is attributed to the *π–π* ^*^ transition characteristic of the aromatic sp^2^‐hybridized carbon domains within the graphitic core [43]. Fluorescence emission is an intrinsic property of CDs. The excitation spectra of AM@CDs, AS@CDs, and AM‐AS@CDs (Figure 2b, recorded at Em = 450 nm) exhibit broad peaks centered around 360 nm, indicating that this wavelength corresponds to the excitation maximum for all three CDs. Meanwhile, Figure 2c presents the optimal emission spectra obtained under this optimal excitation wavelength (360 nm). Under 360 nm UV excitation, all three CDs solutions emit blue fluorescence (Figure S2). Dynamic light scattering (DLS) measurements indicate that the hydrodynamic diameters of AM@CDs, AS@CDs, and AM‐AS@CDs are approximately 4.4, 3.4, and 4.1 nm, respectively (Figure 2d–f). This small size may facilitate their penetration across certain biological barriers [44, 45]. High‐resolution transmission electron microscopy (HR‐TEM) images reveal lattice fringes with a spacing of 0.21 nm, corresponding to the (100) plane of graphitic carbon (Figure 2g).

X‐ray diffraction (XRD) analysis was further employed to investigate the structure of the CDs. The broad XRD peak at around 22° corresponds to the (002) plane of disordered graphitic carbon. No obvious sharp peak is observed at around 43°, an absence that is attributed to the limited stacking order characteristic of ultra‐small carbon dots (Figure 2h). Raman spectra of AM@CDs, AS@CDs, and AM‐AS@CDs (Figure 2i) show a D band at approximately 1350 cm^−1^, originating from sp^3^‐hybridized carbon in the carbon core or surface functional groups. The G band, located around 1580 cm^−1^, is associated with sp^2^‐hybridized carbon. Notably, the intensity ratio of the D band to the G band (*I*_D_/*I*_G_) for the composite AM‐AS@CDs (1.48) is significantly higher than that of the individual AM@CDs (0.58) and AS@CDs (0.78). This result indicates a higher density of structural defects in the composite material [46]. Furthermore, all three types of CDs exhibit negative Zeta potentials (Figure 2j, ranging from −20 to −25 mV), confirming their moderate colloidal stability in aqueous dispersion.

### 2.2 Elucidating the Surface Properties of the CDs

**FIGURE 3:**
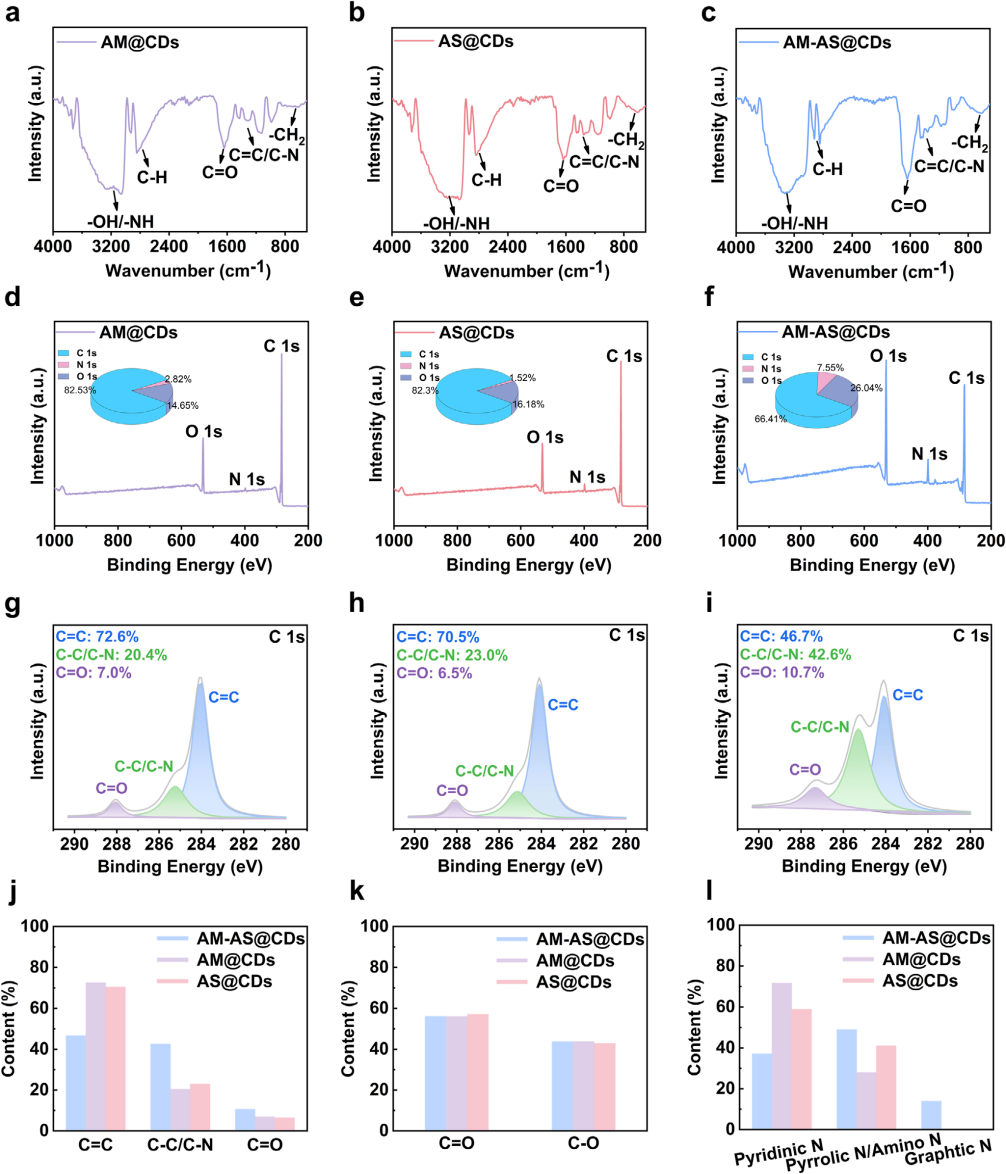
Surface chemical characterization of AM@CDs, AS@CDs, and AM‐AS@CDs. (a–c) FT‐IR spectra, (d–f) XPS survey scans (with elemental composition in atomic %), and (g–i) high‐resolution C 1*s* XPS spectra of AM@CDs, AS@CDs, and AM‐AS@CDs. (j–l) Relative abundances of different chemical states (functional groups) of C, O, and N derived from high‐resolution XPS spectra.

To systematically elucidate the surface chemical characteristics of the CDs, this study employed Fourier transform infrared spectroscopy (FT‐IR) and X‐ray photoelectron spectroscopy (XPS) to characterize the three CD samples. FT‐IR spectra revealed that all CDs exhibited characteristic ─OH/─NH absorption peaks at 3300 cm^−1^, with C─H stretching vibration peaks observed at 2930 cm^−1^ (Figure 3a–c). The absorption signal at 1635 cm^−1^ was attributed to carbonyl functional group vibrations, while the spectral band at 1430 cm^−1^ originated from O─H in‐plane bending or C─N bond stretching vibrations. The prominent peak at 640 cm^−1^ was associated with out‐of‐plane deformation vibrations of aromatic frameworks. The similarity in these spectral features indicates inherent molecular structural relationships among the precursor materials.

XPS elemental quantitative analysis demonstrated distinct compositional differences among the CDs (Figure 3d–f; Figure S3). The composite CDs, AM‐AS@CDs, showed an elemental composition of C (66.41%), N (7.55%), and O (26.04%), while the single‐component CDs, AM@CDs (C: 82.30%, N: 1.52%, O: 16.18%) and AS@CDs (C: 82.53%, N: 2.82%, O: 14.65%), presented significantly different profiles. Notably, the composite CDs exhibited substantially reduced carbon content alongside significantly enhanced incorporation of nitrogen and oxygen heteroatoms. The increase in nitrogen was particularly prominent. Detailed analysis of the C 1*s* fine spectra revealed significantly enhanced peak intensities at 285.2 eV (C─C/C─N bonds) and 288.0 eV (C═O bonds) in the composite CDs (Figure 3g–i) [47]. Quantitative calculations indicated that the relative content of C─C/C─N bonds in AM‐AS@CDs increased by approximately 1.8‐fold compared to single‐component CDs. The proportion of C═O bonds also rose by about 1.5‐fold (Figure 3j–l). This confirms substantial enrichment of nitrogen/oxygen‐containing functional groups in the composite CDs. In contrast, O 1*s* spectral analysis (Figure S4) showed that the three types of CDs maintained nearly identical binding energy positions and peak profiles at 531.3 eV (C═O) and 532.6 eV (C─O). These results indicate no fundamental differences in the chemical environment of oxygen elements among CDs prepared from different precursors. N 1*s* fine spectral analysis further revealed crucial structural information (Figure S5). The composite CDs, AM‐AS@CDs, exhibited three nitrogen configurations, including pyridinic N at 398.7 eV, pyrrolic N/amino N at 399.2 eV, and graphitic N at 400.5 eV. In comparison, single‐component CDs only displayed the first two nitrogen configurations, with no characteristic graphitic N peaks detected. This finding demonstrates that the composite precursor system effectively promotes deeper‐level nitrogen doping into the carbon framework.

Comprehensive analysis indicates that, first, the composite CDs have reduced carbon content and a lower proportion of C─C/C═C bonds. These features suggest a higher defect density in the carbon framework. This is consistent with the XRD and Raman results. Second, the significantly enhanced nitrogen content and diversity of its chemical states in composite CDs, particularly the emergence of graphitic N, reveal the unique advantage of the dual‐precursor system in promoting heteroatom doping. This structural divergence can be attributed to synergistic effects occurring during the thermal transformation of the AM‐AS composite system. These results confirm that the combinatorial strategy of precursor materials can effectively modulate heteroatom doping degree and surface chemical environment. It provides novel insights for the precise control of CDs microstructure and establishes a theoretical foundation for developing carbon‐based materials with specific functionalities.

### 2.3 Elucidation of the Enzyme‐Like Antioxidant Activities of CDs

**FIGURE 4:**
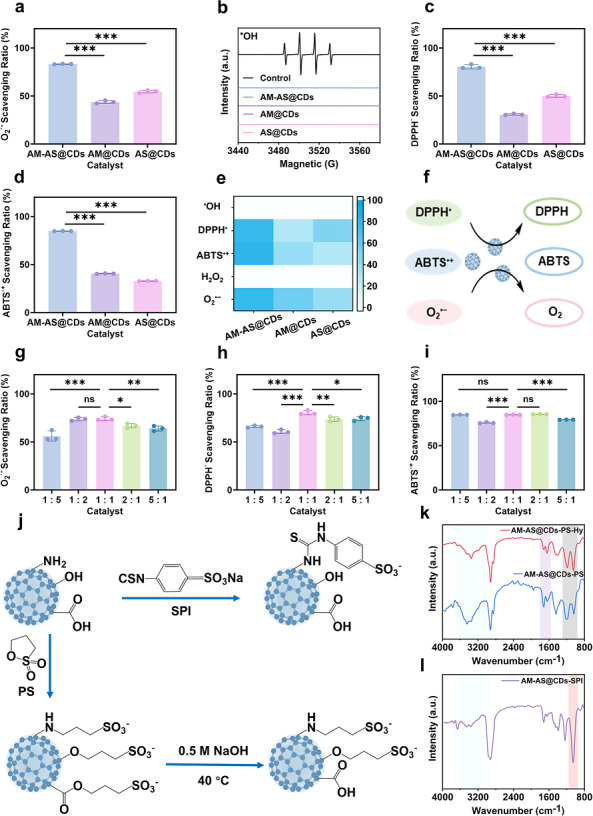
Comprehensive evaluation of enzyme‐mimetic antioxidant activities and catalytic mechanism studies of the CDs. (a) SOD‐like activities of AM@CDs, AS@CDs, and AM‐AS@CDs. (b) EPR spectra demonstrating the absence of hydroxyl radical generation by the CDs. (c), (d) Free radical scavenging capacities assessed via DPPH^•^ (c) and ABTS^•+^ (d) assays. (e), (f) Heatmap of the antioxidant performance of CDs and summary of their radical types. (g–i) SOD‐like activities of composite AM‐AS@CDs synthesized with different precursor mass ratios (AM to AS = 5:1, 2:1, 1:1, 1:2, 1:5), with the optimal 1:1 ratio selected for subsequent studies. (j) Schematic illustration of surface functional group passivation experiments. (k), (l) FT‐IR spectra confirming the successful synthesis of surface‐modified CDs: AM‐AS@CDs‐PS / AM‐AS@CDs‐PS‐Hy (k) and AM‐AS@CDs‐SPI (l). Data are presented as mean ± SD from at least three independent experiments. Statistical comparisons were performed using one‐way ANOVA and *t*‐test; ^*^ *p* < 0.05, ^**^ *p* < 0.01, ^***^ *p* < 0.001; ns, not significant.

To systematically investigate the antioxidative enzyme‐like properties of AM@CDs, AS@CDs, and AM‐AS@CDs, a multi‐dimensional activity assessment was conducted under physiological pH conditions. Their catalytic capabilities toward various ROS were first evaluated. As shown in Figure 4a, the composite CDs, AM‐AS@CDs, exhibited significantly higher SOD‐like activity than the single‐component CDs. To systematically characterize the SOD‐like activity across a wider concentration range, full dose‐response curves were constructed for AM@CDs, AS@CDs, and AM‐AS@CDs. As shown in Figure S6, the superoxide anion scavenging percentage increased in a concentration‐dependent manner for all three nanozymes. From these curves, the specific activities were calculated as 6533 U/mg for AM@CDs, 5944 U/mg for AS@CDs, and 10793 U/mg for AM‐AS@CDs, respectively. The specific activity of AM‐AS@CDs was approximately 1.7‑fold higher than that of the single‐herb‐derived CDs, further confirming the synergistic effect of the AM‐AS composite precursor on enhancing SOD‐mimetic performance. However, none of the three CDs showed detectable CAT‐like activity (Figure S7). Peroxidase (POD) and oxidase (OXD) activity tests confirmed that they do not generate ROS under neutral conditions (Figures S8 and S9). Furthermore, electron paramagnetic resonance (EPR) analysis indicated that none of the CDs produced hydroxyl radicals during the reaction (Figure 4b). These results comprehensively rule out the risk of inducing oxidative stress‐related side effects in their catalytic processes.

In addition to ROS, the scavenging capacity of the CDs toward reactive nitrogen species (RNS) was evaluated using ABTS^+•^ and DPPH^•^ assays (Figure 4c,d). The results showed that AM‐AS@CDs had a significantly higher inhibition rate against DPPH radicals compared to AS@CDs and AM@CDs (Figure 4c). In the ABTS assay, AM‐AS@CDs also markedly suppressed the formation of ABTS^+•^ (Figure 4d). In summary, AM‐AS@CDs exhibited superior scavenging efficiency across multiple antioxidant indices, underscoring their excellent comprehensive antioxidant performance. This observation is consistent with previous reports on multi‐component herbal systems [48]. Collectively, these results support the advantage of nanoscale integration of complementary components and highlight the considerable potential of AM‐AS@CDs for the prevention and treatment of I/R‐induced AKI (Figure 4e,f).

Given the excellent antioxidant capacity of the composite CDs AM‐AS@CDs, we further investigated the effect of precursor ratios. CDs with different mass ratios of AM to AS (5:1, 2:1, 1:1, 1:2, 1:5) were synthesized for this purpose. As shown in Figure 4g–i, all composite CDs with different ratios displayed good antioxidant activity. The results indicated that varying the precursor ratio did not lead to a significant enhancement in activity. The cytotoxicity, antioxidant capacity, and anti‐apoptotic activity of AM‐AS@CDs prepared with these different precursor ratios were further evaluated in NRK‐52E cells (Figures S10a–e and S11). The results from these cellular assays were consistent with the observed antioxidant activity trends. The 1:1 ratio yielded optimal performance among all tested formulations. Due to its superior catalytic output and consistent uniformity, it was selected for all subsequent studies.

To gain deeper mechanistic insight into the catalysis of AM‐AS@CDs at the 1:1 ratio, a series of surface functional group masking experiments were systematically performed. Initially, to test the hypothesis that surface groups regulate the SOD‐like activity, selective chemical modifications were applied to AM‐AS@CDs (Figure 4j). The surface amino groups were passivated via reaction with 4‐sulfophenyl isothiocyanate (SPI), yielding AM‐AS@CDs‐SPI. FTIR characterization confirmed successful modification (Figure 4k). Specifically, the ─NH peak at 3300 cm^−1^ weakened significantly, while a new characteristic peak appeared at 1060 cm^−1^, assigned to the stretching vibrations of the sulfonate group (O═S═O).

To further investigate the role of oxygen‐containing functional groups, AM‐AS@CDs were simultaneously modified using 1,3‐propanesultone (PS) to passivate amino, carboxyl, and hydroxyl groups, resulting in AM‐AS@CDs‐PS. The FTIR spectra confirmed the multi‐group passivation (Figure 4l). The ─OH/─NH absorption peak at 3300 cm^−1^ was significantly attenuated, while the carboxylate peak at 1635 cm^−1^ shifted to 1700 cm^−1^, indicating ester bond formation. Additionally, characteristic absorption peaks for ─SO_3_^−^ appeared at 1200 and 1040 cm^−1^. Subsequently, alkaline hydrolysis of AM‐AS@CDs‐PS was conducted to restore the carboxyl groups, producing AM‐AS@CDs‐PS‐Hy. The FTIR spectra of AM‐AS@CDs‐PS‐Hy showed the characteristic ester peak at 1700 cm^−1^ shifting back to 1635 cm^−1^ (Figure 4l), verifying the successful recovery of the original carboxyl groups.

Following the successful synthesis and characterization of this series of materials, their superoxide anion scavenging capacity was uniformly evaluated using the WST‐1 method. The activity assay results (Figure S12) revealed that compared to the pristine AM‐AS@CDs, AM‐AS@CDs‐SPI (with only amino groups passivated) exhibited a significant decrease in scavenging activity, highlighting the critical role of amino groups. When amino, carboxyl, and hydroxyl groups were all passivated (AM‐AS@CDs‐PS), the activity dropped to the lowest level. After the restoration of carboxyl groups (AM‐AS@CDs‐PS‐Hy), a partial recovery in activity was observed, indicating a positive contribution of carboxyl groups. Notably, the activity of AM‐AS@CDs‐PS‐Hy was lower than that of AM‐AS@CDs‐SPI, demonstrating that hydroxyl groups also participate synergistically in the activity regulation. These results systematically illustrate the individual contributions and synergistic mechanism of the surface amino, carboxyl, and hydroxyl groups on AM‐AS@CDs toward their SOD‐like activity.

Beyond these surface functional groups, the bulk structural features of AM‐AS@CDs provide further mechanistic insight. The excellent SOD‐like activity of AM‐AS@CDs correlates with three interrelated structural characteristics: (i) significantly higher nitrogen doping (7.55%) compared to AM@CDs (1.52%) and AS@CDs (2.82%); (ii) a higher Id/Iɢ ratio (1.48 vs. 0.58 and 0.78), indicating increased defect density; and (iii) the emergence of graphitic N in the N 1s XPS spectra, which is absent in the single‐precursor CDs. These features are not independent. Enhanced nitrogen doping, particularly graphitic N, disrupts the sp^2^ carbon network, generating more defect sites and providing additional anchoring points for surface functional groups such as amino, carboxyl, and hydroxyl groups. Theoretical and experimental studies have shown that nitrogen‐doped carbon dots exhibit a lower energy barrier for superoxide anion scavenging and that defects can act as active centers for electron transfer [42, 49]. Thus, the synergy of higher nitrogen doping and higher defect density underpins the superior SOD‐like activity of AM‐AS@CDs. Collectively, these findings highlight that the rational combination of herbal precursors can effectively engineer both the bulk defects and surface chemistry of carbon dots, offering a promising strategy for developing high‐performance antioxidant nanomaterials.

### 2.4 AM‐AS@CDs Mitigate Cellular Oxidative Stress and Apoptosis

AM‐AS@CDs exhibit superior SOD‐mimetic activity and originate from a composite herbal system (AM‐AS). These features provide a basis for exploring novel therapeutic functions and mechanisms. Mitochondrial oxidative stress and excessive ROS are hallmarks of I/R injury [50, 51]. To systematically evaluate the antioxidant potential and cytoprotective efficacy of AM@CDs, AS@CDs, and AM‐AS@CDs, NRK‐52E cells were exposed to graded concentrations (0–100 µg/mL) of the respective CDs for 24 h. The results showed no significant loss of viability (Figure S10a,f,g). Based on this range, preliminary dose‐screening experiments were performed for AM‐AS@CDs using 15, 30, 60, and 90 µg/mL. Among these concentrations, 30 µg/mL showed the strongest antioxidant and anti‐apoptotic effects and was used thereafter (Figure S13). The same concentration was then applied to AM@CDs and AS@CDs for direct comparison.

**FIGURE 5:**
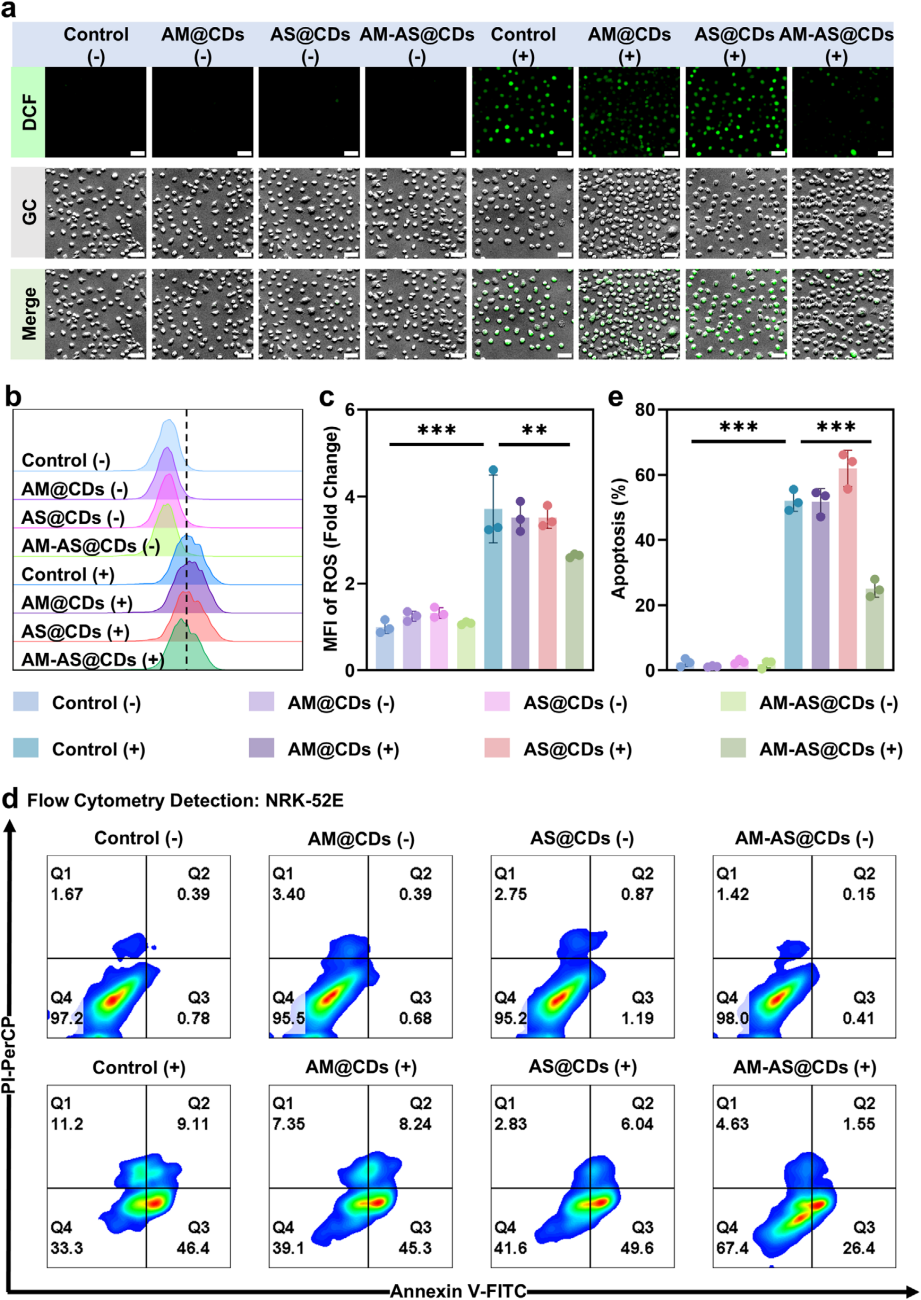
In vitro antioxidant and anti‐apoptotic activities of AM‐AS@CDs. (a) Fluorescence micrographs of intracellular ROS in NRK‐52E cells under indicated treatments (scale bar = 50 µm). (b) Flow cytometric analysis of ROS levels in NRK‐52E cells. (c) Quantification of relative ROS levels normalized to the control group (*n* = 3). (d), (e) Representative flow‐cytometry plots and quantitative analysis of apoptosis in NRK‐52E cells (*n* = 3). Cells in the Q2 (Annexin V^+^ PI^+^) and Q3 (Annexin V^+^ PI^−^) quadrants were defined as late and early apoptotic cells, respectively. Cells in these two quadrants were considered apoptotic. “+” denotes H_2_O_2_‐stimulated injury model, “‐” denotes untreated control. MFI, mean fluorescence intensity. Data are presented as mean ± SD from at least three independent experiments. Statistical comparisons were performed using one‐way ANOVA and *t*‐test; ^*^ *p* < 0.05, ^***^ *p* < 0.001; ns, not significant.

An oxidative insult was next modeled by H_2_O_2_ to recapitulate the ROS burst observed after I/R. The DCFH‐DA fluorescence probe was used to assess intracellular ROS levels. Compared with the AM@CDs and AS@CDs groups, AM‐AS@CDs significantly reduced superoxide anion levels (Figure 5a). Flow cytometric quantification further demonstrated that the H_2_O_2_‐induced ROS was effectively suppressed by AM‐AS@CDs, but not by the CDs derived from single herbs (Figure 5b,c). For further validation, we included N‐acetylcysteine (NAC), a clinically used antioxidant for acute kidney injury, as an external benchmark. AM‐AS@CDs exhibited stronger ROS‐scavenging capacity than NAC under the same experimental conditions (Figure S14a,b). Because oxidative stress usually culminates in irreversible damage and cell death, Annexin V‐FITC/PI double staining was employed to appraise anti‐apoptotic capacity. Among the three CDs, AM‐AS@CDs most effectively attenuated apoptosis triggered by H_2_O_2_ (Figure 5d,e). We further found that AM‐AS@CDs exhibited stronger anti‐apoptotic effects compared to NAC in supplementary experiments (Figure S14c,d). To extend these findings beyond the NRK‐52E cell line, experiments were performed in human HK‐2 cells and primary mouse renal tubular epithelial cells (RTECs). In both models, AM‐AS@CDs showed low cytotoxicity under normal conditions (Figure S10h,i). They also showed strong antioxidant and anti‐apoptotic effects under H_2_O_2_‐induced oxidative stress (Figures S15 and S16). The consistent responses across immortalized and primary cells suggest that the protective effects are not cell line‐specific. Instead, they are broadly applicable to renal epithelial cells. Collectively, AM‐AS@CDs provide superior protection against H_2_O_2_‐induced injury compared with the single‐herb‐derived CDs.

### 2.5 In Vivo Attenuation of Renal I/R Injury by AM‐AS@CDs

**FIGURE 6:**
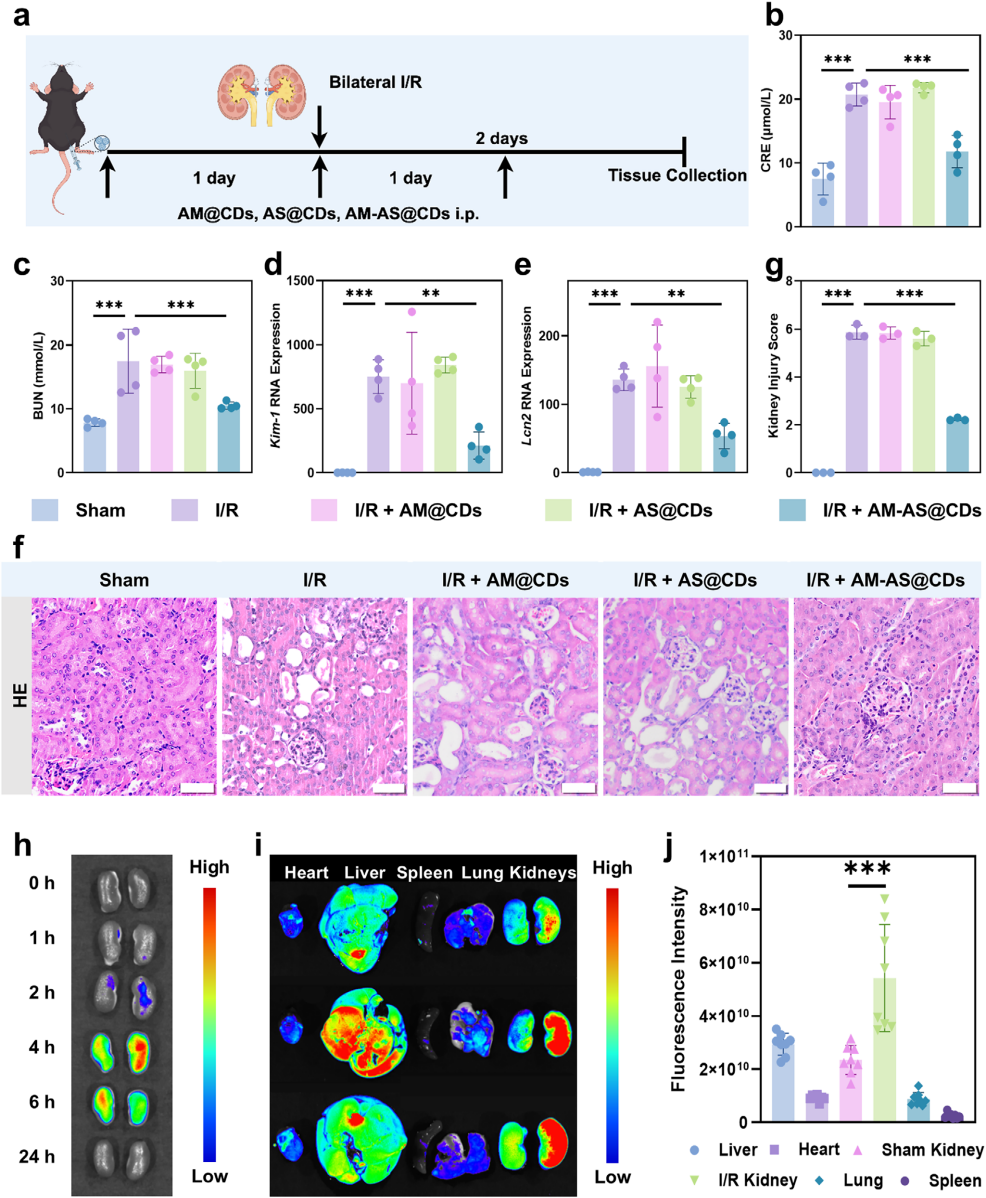
Renoprotective efficacy of AM‐AS@CDs after I/R and their distribution. (a) Schematic timeline illustrating the prophylactic‐therapeutic regimen for different CDs treatment groups. (b), (c) Functional assessment of kidney injury 48 h post‐reperfusion (*n* = 4). CRE, creatinine; BUN, blood urea nitrogen. (d), (e) Renal mRNA expression levels of *Kim‐1* (d) and *Lcn2* (e) in each group (*n* = 4). (f) Representative H&E‐stained histological images (scale bar = 50 µm). (g) Damage scores of kidney tissues (*n* = 3). (h) Time‐dependent accumulation of AM‐AS@CDs in the kidney. (i) Ex vivo fluorescence images of major organs excised from I/R mice 4 h after AM‐AS@CDs administration. (j) Quantitative analysis of fluorescence intensity in the organs (*n* = 8). Data are presented as mean ± SD from at least three independent experiments. Statistical comparisons were performed using one‐way ANOVA and *t*‐test; ^**^ *p* < 0.01, ^***^ *p* < 0.001.

AM‐AS@CDs exhibit superior antioxidant and anti‐apoptotic activities in H_2_O_2_‐injured cells. Based on these findings, we systematically compared the therapeutic efficacies of AM@CDs, AS@CDs, and AM‐AS@CDs in a murine model of bilateral renal I/R‐induced AKI. A prophylactic‐therapeutic dosing regimen was adopted (Figure 6a). After 48 h of reperfusion, serological analyses were performed. Serum creatinine (CRE) and blood urea nitrogen (BUN) levels were markedly reduced in the I/R + AM‐AS@CDs group. No significant changes were observed in the I/R + AM@CDs or I/R + AS@CDs groups (Figure 6b,c). Molecularly, mRNA levels of early tubular injury biomarkers *Kim‐1* and *Lcn2* were significantly down‐regulated following AM‐AS@CDs treatment (Figure 6d,e). At the histological level, H&E staining showed extensive tubular dilation, vacuolar degeneration, and brush‐border loss in the I/R, I/R + AM@CDs, and I/R + AS@CDs groups. These pathological alterations were substantially alleviated in the AM‐AS@CDs‐treated cohort, as evidenced by a lower tubular necrosis score (Figure 6f,g).

The above results indicate that the prophylactic‐therapeutic regimen provides sustained renal protection. This makes it particularly suitable for clinical scenarios such as kidney transplantation or partial nephrectomy. However, in many clinical situations, prophylactic administration is not feasible. Therefore, we further evaluated the therapeutic efficacy of AM‐AS@CDs when administered after reperfusion (Figure S17a). The results showed that post‐reperfusion treatment with AM‐AS@CDs exerted a protective effect against renal injury (Figure S17b–g). Importantly, this indicates that AM‐AS@CDs remain effective under both regimens, highlighting their broad therapeutic potential for AKI. Further analysis revealed that the prophylactic‐therapeutic regimen conferred superior protection compared with post‐reperfusion treatment (Figure S17b–g). Therefore, to better investigate the therapeutic mechanisms of AM‐AS@CDs, the prophylactic‐therapeutic strategy was selected for subsequent experiments.

Compared with the PBS cohort, H&E staining showed that the intact tissue structures in major organs, including the heart, liver, spleen, lungs, and kidneys. No obvious inflammatory infiltration or necrosis was observed (Figure S18a). Neither body weight nor the kidney‐to‐body weight ratio exhibited statistically significant deviation (Figure S18b,c). Serum biochemical indices of hepatic and renal function (ALT, AST, CRE, and BUN) remained unchanged (Figure S18d–g). These results indicate excellent biocompatibility of AM‐AS@CDs.

To rigorously evaluate the therapeutic potential of AM‐AS@CDs against renal I/R injury, we delineated its biodistribution in mice kidneys. Due to the risk of false‐positive signals from probe detachment [52, 53], we exploited the intrinsic optical property of AM‐AS@CDs (Figure S2). This enabled direct ex vivo imaging of kidneys harvested at serial time points after intraperitoneal (i.p.) administration. AM‐AS@CDs was enriched in the kidney 2 h after injection and remained stable for at least 4 h (Figure 6h). These results indicate rapid and sustained renal enrichment of AM‐AS@CDs.

Next, we interrogated whether I/R injury modulates the distribution profile of AM‐AS@CDs. Following 25 min of left renal ischemia and 44 h of reperfusion, mice were injected with AM‐AS@CDs and imaged 4 h later. Ex vivo organ imaging demonstrated predominant accumulation in the liver and kidneys, with minimal signals in the lungs, spleen, and heart (Figure 6i). Notably, fluorescence intensity in I/R kidneys was significantly higher than in contralateral normal kidneys (Figure 6j). This increased uptake is likely attributable to increased vascular permeability and inflammatory chemotaxis. Collectively, AM‐AS@CDs have both renal accumulation and good biocompatibility, and can significantly improve renal I/R injury in mice. This effect is associated with reduced tissue edema, inhibition of tubular injury, and restoration of renal function.

### 2.6 AM‐AS@CDs Attenuate Immune Cell Recruitment and Infiltration in Peripheral Blood and Kidneys After I/R

**FIGURE 7:**
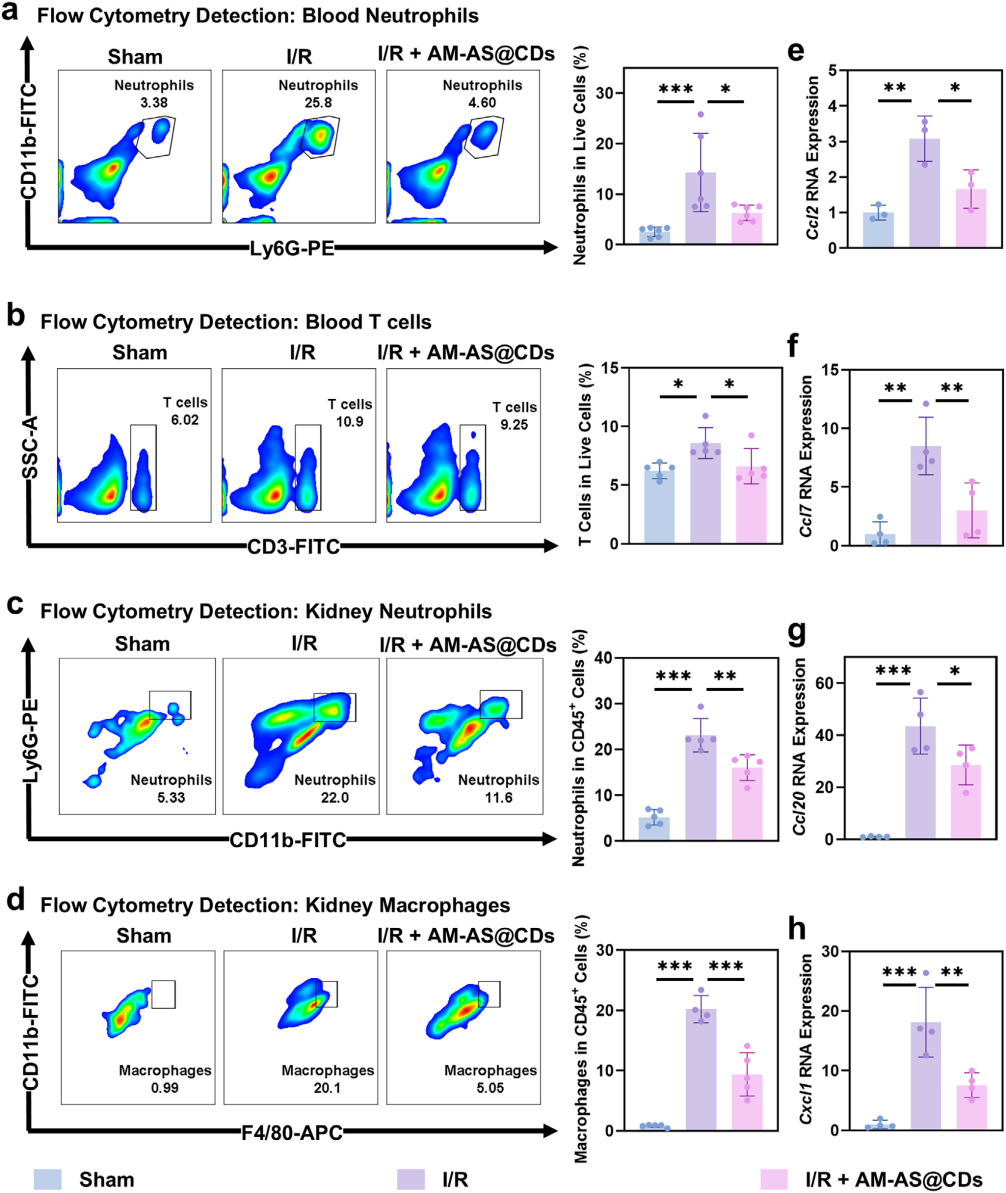
AM‐AS@CDs attenuate immune cell infiltration in peripheral blood and kidneys of mice subjected to renal I/R injury. (a) Representative flow cytometric plots and quantitative analysis of circulating neutrophils (*n* = 6). (b) Representative flow cytometric plots and quantitative analysis of circulating T lymphocytes (*n* = 5). (c) Representative flow cytometric plots and quantitative analysis of renal neutrophils (*n* = 5). (d) Representative flow cytometric plots and quantitative analysis of renal macrophages (*n* = 4). (e–h) The results of qRT‐PCR of chemokines *Ccl2* (*n* = 3), *Ccl7* (*n* = 4), *Ccl20* (*n* = 4) and *Cxcl1* (*n* = 4) in different treatment groups. Data are presented as mean ± SD from at least three independent experiments. Statistical comparisons were performed using one‐way ANOVA and *t*‐test; ^*^ *p* < 0.05, ^**^ *p* < 0.01, ^***^ *p* < 0.001.

Post‐ischemic immune cell recruitment constitutes a pivotal initiator of renal inflammation following I/R injury [54]. Neutrophils and other innate immune cells amplify tissue damage via the release of pro‐inflammatory cytokines and chemokines that further recruit circulating leukocytes to the injured kidney. To delineate the immunomodulatory capacity of AM‐AS@CDs, we performed high‐dimensional flow cytometry on peripheral blood and enzymatically digested renal tissue. AM‐AS@CDs administration markedly reduced the frequencies of circulating neutrophils (Ly6G^+^CD11b^+^) and total T cells (CD3^+^) (Figure 7a,b) without altering the CD4^+^/CD8^+^ T‐cell ratio (Figure S19). Consistently, renal infiltration of both neutrophils (Ly6G^+^CD11b^+^) and macrophages (F4/80^+^CD11b^+^) was significantly diminished (Figure 7c,d).

Subsequently, qRT‐PCR revealed that AM‐AS@CDs down‐regulated the transcription of key chemokine genes, including *Ccl2*, *Ccl7*, *Ccl20*, and *Cxcl1* (Figure 7e–h). Additionally, Western Blot (WB) analysis showed that AM‐AS@CDs treatment significantly reduced the protein levels of NF‐κB. It also suppressed the transcription of core pro‐inflammatory cytokines, including TNF‐α and IL‐1β (Figure S20). Collectively, these data indicate that AM‐AS@CDs attenuate I/R‐induced renal injury by disrupting the chemokine‐receptor axis. This effect limits immune cell extravasation and the ensuing inflammatory cascade.

### 2.7 AM‐AS@CDs Attenuate Renal Oxidative Stress Following I/R Injury

**FIGURE 8:**
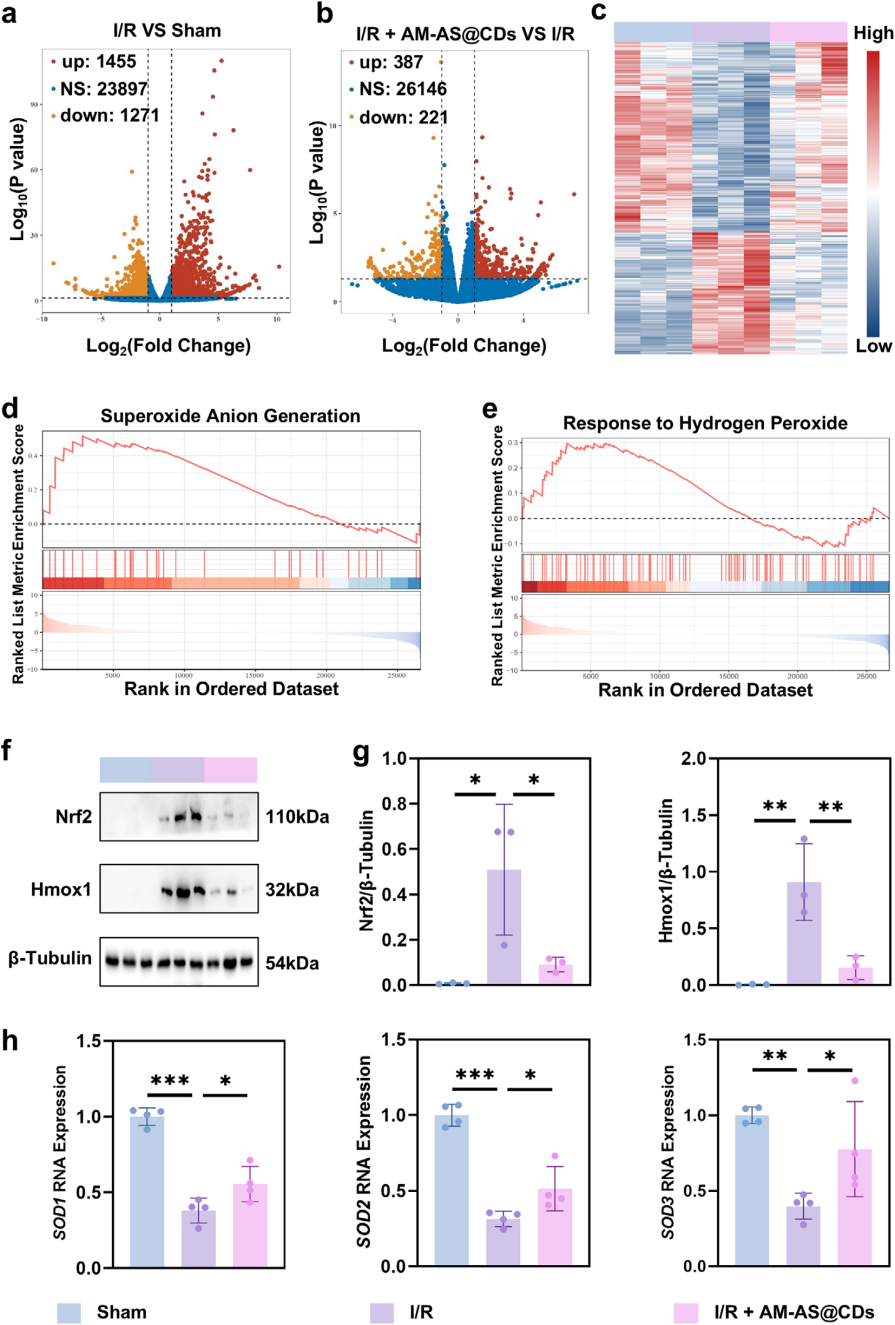
AM‐AS@CDs attenuate renal oxidative stress in I/R‐challenged mice. (a), (b) Volcano plots of DEGs: I/R vs. Sham (a) and I/R + AM‐AS@CDs vs. I/R (b). (c) Heat map of DEGs across the three treatments. (d), (e) GSEA of the Sham group and the I/R group. (f), (g) WB analysis of Nrf2 and Hmox1 expression in each group (*n* = 3). (h) The results of qRT‐PCR of *SOD1*, *SOD2*, and *SOD3* in different treatment groups (*n* = 4). Data are presented as mean ± SD from at least three independent experiments. Statistical comparisons were performed using one‐way ANOVA and *t*‐test; ^*^ *p* < 0.05, ^**^ *p* < 0.01, ^***^ *p* < 0.001.

To further delineate the molecular mechanisms underlying the renoprotective effects of AM‐AS@CDs in renal I/R injury, total RNA was extracted from mouse kidneys 48 h after reperfusion. Library preparation and high‐throughput sequencing were then performed. Pearson correlation analysis revealed correlation coefficients > 0.92 across all samples, indicating high inter‐sample concordance (Figure S21). Principal component analysis (PCA) clearly segregated the experimental groups, demonstrating dataset reliability and effective control of batch effects (Figure S22). Differential expression analysis identified 1455 up‐regulated and 1271 down‐regulated genes in I/R vs. Sham kidneys (|log_2_FC| ≥ 1, p < 0.05; Figure 8a). In the AM‐AS@CDs treatment group, 387 genes were upregulated, and 221 genes were downregulated compared with the I/R group (Figure 8b). Heat‐map visualization of these differentially expressed genes (DEGs) revealed distinct transcriptional profiles among groups with high intra‐group reproducibility (Figure 8c). Gene set enrichment analysis (GSEA) revealed significant enrichment of gene sets related to oxidative stress and ROS generation in the I/R kidneys (Figure 8d,e). WB further confirmed that I/R evoked compensatory activation of the Nrf2/HO‐1 pathway. In contrast, AM‐AS@CDs treatment restored this pathway to baseline levels (Figure 8f,g). This result indicates that AM‐AS@CDs can re‐establish redox homeostasis disrupted by I/R injury. qRT‐PCR analyses revealed that AM‐AS@CDs significantly elevated the transcript levels of *SOD1*, *SOD2, SOD3, GPX4*, and *CAT* (Figure 8h; Figure S23). These results indicate an antioxidant mechanism of AM‐AS@CDs. They suppress ROS accumulation and preserve endogenous antioxidant gene expression.

### 2.8 Transcriptomic Dissection of the Renoprotective Network Governed by AM‐AS@CDs

**FIGURE 9:**
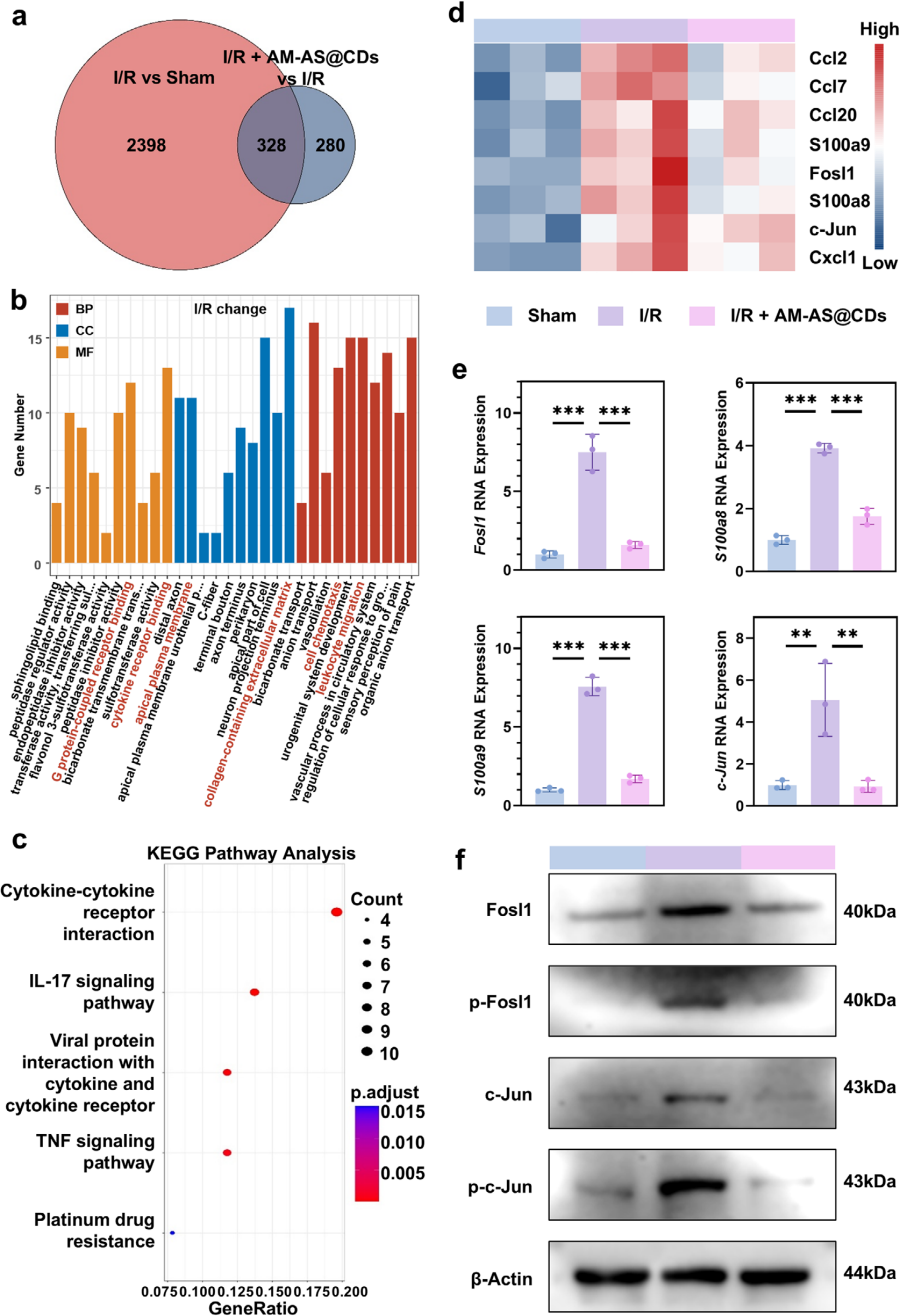
RNA‐seq elucidates the biological mechanisms underlying I/R injury and AM‐AS@CDs intervention. (a) Venn diagram illustrating the intersection of DEGs identified in I/R vs. Sham and I/R + AM‐AS@CDs vs. I/R comparisons. (b) GO enrichment analysis of the intersected DEGs (BP: Biological Process; CC: Cellular Component; MF: Molecular Function). (c) KEGG pathway enrichment for genes up‐regulated by I/R within the DEG intersection. (d) Expression heat map of selected genes across the three groups. (e) The results of qRT‐PCR of *Fosl1*, *S100a8*, *S100a9*, and *c‐Jun* in different treatment groups (*n* = 3). (f) Representative WB analysis of Fosl1, p‐Fosl1, c‐Jun, and p‐c‐Jun expression in each group. Data are presented as mean ± SD from at least three independent experiments. Statistical comparisons were performed using one‐way ANOVA and *t*‐test; ^**^ *p* < 0.01, ^***^ *p* < 0.001.

To pinpoint the core regulatory network mediating the protective effects of AM‐AS@CDs, we intersected the DEGs derived from Sham vs. I/R and I/R + AM‐AS@CDs vs. I/R comparisons. This analysis yielded 328 concordant genes with consistent directional changes (Figure 9a). Gene Ontology (GO) enrichment revealed significant over‐representation of “cytokine receptor binding”, “leukocyte migration”, and “positive regulation of inflammatory response” (Figure 9b). Kyoto Encyclopedia of Genes and Genomes (KEGG) enrichment further revealed that genes up‐regulated by I/R and reversed by AM‐AS@CDs were predominantly involved in the cytokine‐cytokine receptor interaction, IL‐17 signaling, and TNF signaling pathways (Figure 9c). These results are consistent with our flow‐cytometry data on immune cell infiltration.

Focusing on the chemokine‐inflammation axis, heat‐map analysis demonstrated that AM‐AS@CDs markedly suppressed the expression of chemokines (Ccl2, Ccl7, Ccl20, and Cxcl1), as well as the S100 proteins S100a8 and S100a9 (Figure 9d). Notably, Fos‐related antigen 1 (Fosl1/Fra‐1) and Jun Proto‐Oncogene (c‐Jun) exhibited significantly enhanced transcriptional activity after I/R. This increase was attenuated by AM‐AS@CDs treatment (Figure 9d). These changes were independently validated by qRT‐PCR (Figure 9e). WB analyses corroborated these findings at the protein level: I/R elevated c‐Jun, phospho‐c‐Jun (p‐c‐Jun), Fosl1, and phospho‐Fosl1 (p‐Fosl1), whereas AM‐AS@CDs reversed these increases (Figure 9f). Fosl1 and c‐Jun, members of the Fos and Jun families, constitute essential components of the transcription factor activator protein‐1 (AP‐1) complex that orchestrates diverse cellular processes. AP‐1 initiates inflammatory responses and recruits immune cells by directly binding to the promoters of chemokine and cytokine genes and activating their transcription and expression [55]. A rise in the AP‐1 constituents Fosl1 and c‐Jun nucleates the active dimer, unleashing downstream leukocyte infiltration and propagating the inflammatory cascade [56]. Collectively, these data indicate that AM‐AS@CDs suppress c‐Jun/Fosl1/AP‐1 signaling. This effect limits chemokine‐mediated leukocyte infiltration and renal inflammatory injury.

## 3 Conclusions

In summary, this study demonstrates a novel strategy of integrating two complementary TCM herbs into a CDs precursor. The synthesized AM‐AS@CDs effectively combine the therapeutic profile of natural medicines with the potent antioxidant function of nanozymes. They exhibit superior renoprotective efficacy compared with CDs derived from single herbs. Within a biological context, AM‐AS@CDs display excellent antioxidant activity and renal accumulation. Mechanistically, they attenuate the amplification of AP‐1‐dependent inflammation by downregulating the expression and phosphorylation of transcription factors Fosl1 and c‐Jun. This effect provides significant protection against I/R‐induced AKI. This work confirms that herb‐composite‐derived CDs retain potent bioactivity and provides a systematic evaluation of their therapeutic potential. Overall, the findings offer novel insights and a material platform for developing functionalized nanozymes from natural medicinal resources to treat I/R‐related disorders.

## 4 Experimental Section

### 4.1 Reagents and Materials

AM and AS were purchased from Nanjing Tongren Hospital Pharmaceutical Co., Ltd. H_2_O_2_ (3% in water) was sourced from Aladdin (Shanghai, China). FITC anti‐mouse CD11b, PE anti‐mouse Ly6G, FITC anti‐mouse CD3, APC anti‐mouse F4/80, PE anti‐mouse CD4, and APC anti‐mouse CD8 were purchased from eBioscience; PerCP anti‐mouse CD45 was obtained from BioLegend. Type I collagenase was sourced from MedChemExpress. The Cell Counting Kit‐8 and ROS Assay Kit were procured from Beyotime Chemical Reagent Co., Ltd. (Shanghai, China). The Annexin V‐Alexa Fluor 647/PI apoptosis detection kit was purchased from Elabscience Biotechnology Co., Ltd. (Wuhan, China). RIPA lysis buffer and BCA protein assay kit were obtained from Beyotime Chemical Reagent Co., Ltd. (Shanghai, China). Protease inhibitors were purchased from APExBIO, and phosphatase inhibitors were obtained from KeyGEN BioTECH (Nanjing, China). The CRE content detection kit (sarcosine oxidase method) was procured from Jiangsu Aidisheng Biological Technology Co., Ltd., and the BUN test kit (urease method) was obtained from Nanjing Jiancheng Bioengineering Institute (Jiangsu, China). The Aspartate Aminotransferase (AST/GOT) Activity Assay Kit and Alanine Aminotransferase (ALT/GPT) Activity Assay Kit (E‐BC‐K235‐M) were purchased from Elabscience Biotechnology Co., Ltd. (Wuhan, China). Primary antibodies used for WB were as follows: Fosl1, Phospho‐c‐Jun (Thr91), c‐Jun, and Nrf2 were purchased from Bioworld Biotech Co., Ltd. (Nanjing, China); NF‐κB and phosphor‐Fosl1(Ser265) were obtained from Cell Signaling Technology; Hmox1, β‐Tubulin, and β‐Actin were purchased from Proteintech.

### 4.2 Preparation of AM‐AS@CDs, AM@CDs, and AS@CDs

#### 4.2.1 AM‐AS@CDs

Initially, AM and AS roots were separately ground into fine powders using a ball mill. The powders were then homogeneously mixed at a 1:1 mass ratio. An appropriate amount of ultrapure water was added as solvent, and the mixture was uniformly dispersed via ultrasonication. The resulting dispersion was transferred to a reaction autoclave and subjected to hydrothermal reaction at 180°C for 6 h. After reaction completion, centrifugation was performed to remove precipitates and collect the supernatant. This supernatant was sequentially filtered through 0.22 µm aqueous‐phase and organic‐phase membranes, followed by further purification using ultrafiltration centrifuge tubes. The purified solution was loaded into dialysis bags and dialyzed against ultrapure water for 3 days. Finally, the dialyzed solution was lyophilized to obtain AM‐AS@CDs solid powder.

#### 4.2.2 AM@CDs and AS@CDs

Parallel procedures were employed for AM@CDs and AS@CDs synthesis, with the sole modification being the exclusive employment of single‐component raw materials (either AM or AS) during the initial grinding stage. All subsequent steps‐including dispersion, hydrothermal reaction, separation, filtration, ultrafiltration purification, dialysis, and lyophilization‐remained identical to the AM‐AS@CDs protocol.

### 4.3 Material Characterization

The morphology of the samples was characterized using transmission electron microscopy (TEM) on a Tecnai 12 microscope (Philips, Netherlands) operated at 120 kV and a JEM‐2100 microscope (JEOL, Japan) operated at 200 kV. XRD patterns were recorded on a Rigaku Ultima III diffractometer (Japan) with Cu Kα radiation and a Bruker D8 advance diffractometer (Germany). UV–vis absorption spectra were acquired using a Cary UV‐‑vis 100 spectrophotometer (Agilent Technologies, USA) and a SpectraMax M2e microplate reader (Molecular Devices, USA). Surface chemical states were analyzed by XPS using a PHI 5000 Versa Probe system (Ulvac‐Phi, Japan) and a K‐Alpha spectrometer (Thermo Fisher Scientific, USA).

### 4.4 POD‐Like and OXD‐Like Activity Assays

POD‐like activity was evaluated by monitoring the oxidation of TMB at 652 nm. The reaction mixture (1 mL) contained 30 µL of AM‐AS@CDs (1 mg/mL), 50 µL of TMB (10 mm), 100 µL of H_2_O_2_ (10 mm), and 820 µL of PBS buffer (10 mm, pH 7.0). After incubation at 25°C for 20 min, the absorbance was measured.

OXD‐like activity was assessed under identical conditions except that H_2_O_2_ was omitted. The reaction system consisted of 30 µL of AM‐AS@CDs (1 mg/mL), 50 µL of TMB (10 mm), and 920 µL of PBS buffer (10 mm, pH 7.0).

### 4.5 SOD‐ and Cat‐Like Catalytic Activity Assay

#### 4.5.1 Measurement of SOD‐Like Activity

The SOD‐like activity of AM‐AS@CDs, AM@CDs, and AS@CDs nanozymes was evaluated using a WST‐1 assay kit. All samples were prepared from a 1 mg/mL stock solution and diluted as needed for each assay under physiological pH conditions. In brief, 20 µL of sample solution was mixed with 200 µL of WST‐1 working solution and 20 µL of enzyme working solution in a 96‑well plate. After incubation at 37 °C for 20 min, the absorbance at 450 nm was recorded. Blank controls included: Blank 1 (ddH_2_O instead of sample), Blank 2 (sample without enzyme working solution), and Blank 3 (ddH_2_O without enzyme working solution). The inhibition rate was calculated as: Inhibition (%) = [1 – (OD_sample_—OD_Blank2_) / (OD _Blank1_ – OD _Blank3_)] × 100. Specific activity (U/mg) was determined from the full dose‐response curve, where one unit (U) is defined as the amount of nanozyme required to achieve 50% inhibition under the assay conditions. All measurements were performed in triplicate.

#### 4.5.2 Measurement of CAT‐Like Activity

The CAT‐like activity of AM‐AS@CDs, AM@CDs, and AS@CDs nanozymes was measured by monitoring the amount of O_2_ generated as well as the elimination of H_2_O_2_. First, the nanozyme sample was mixed with H_2_O_2_ (5 mm). Immediately after that, the amount of O_2_ was recorded using a dissolved oxygen meter at intervals of 300 s.

### 4.6 Primary RTECs Isolation and Culture

Primary mouse RTECs were isolated from approximately 1‐week‐old mice. Kidneys were collected under sterile conditions and minced, followed by filtration through 50‐mesh and 150‐mesh sieves. The collected tissue was digested with type I collagenase (1 mg/mL) at 37°C for 25 min with gentle agitation. After centrifugation and washing with PBS, the cells were resuspended in DMEM/F12 medium supplemented with 10% fetal bovine serum, 1% penicillin‐streptomycin, 1% ITS, and 10 ng/mL mouse epidermal growth factor. Cells were seeded in culture dishes, and the medium was replaced after 24 h. Cells were incubated in a humidified incubator at 37°C with 5% CO_2_.

### 4.7 Cell Lines

Human RTECs line (HK‐2) and rat proximal RTECs (NRK‐52E) were obtained from the Institute of Biochemistry and Cell Biology, Shanghai Institutes for Biological Sciences, Chinese Academy of Sciences. HK‐2 cells were cultured in DMEM complete medium (comprising 90% DMEM, 10% fetal bovine serum, 100 U/mL penicillin, and 0.1 mg/mL streptomycin). NRK‐52E cells were maintained in DMEM/F12 complete medium (containing 90% DMEM, 10% fetal bovine serum, 100 U/mL penicillin, and 0.1 mg/mL streptomycin). All cells were incubated in a humidified incubator at 37°C with 5% CO_2_.

### 4.8 Intracellular ROS Detection

RTECs, HK‐2, and NRK‐52E cells were seeded into 12‐well plates and cultured overnight in complete medium. Subsequently, 30 µg/mL of corresponding CDs was added to different wells for a 2‐h pre‐incubation. NAC (1 mm) was included as an external antioxidant control in independent experiments for benchmarking purposes. Cells were then exposed to oxidative stress by treatment with 100 µm H_2_O_2_ (for HK‐2 cells) and 200 µm H_2_O_2_ (for NRK‐52E cells and RTECs). After a 1‐h incubation with H_2_O_2_, cells were incubated with a 1:1000 dilution of DCFH‐DA fluorescent probe for 20 min. Following incubation, cells were washed three times with PBS. Fluorescence intensity was observed and imaged using the APEXVIEW APX100 Desktop Fluorescence Microscope. Additionally, cells treated under the same conditions were analyzed by flow cytometry for quantitative assessment of ROS levels.

### 4.9 Apoptosis Assessment by Annexin V‐Alexa Fluor 647/PI Staining

Cells were processed as described above. Finally, cells were treated with 100 µm H_2_O_2_ (for HK‐2 cells) and 200 µm H_2_O_2_ (for NRK‐52E cells and RTECs) for 24 h, with or without 30 µg/mL of the corresponding CDs. NAC (1 mm) was included as a control in independent experiments for benchmarking purposes. After treatment, the culture medium was removed, and the cells were collected and washed with PBS. Cells were then stained with Annexin V‐Alexa Fluor 647/PI and analyzed by flow cytometry to assess apoptosis.

### 4.10 Animal Studies

Male C57BL/6J mice (6–8 weeks old) were obtained from Huachuang Sino Pharmaceutical Technology Co., Ltd. (Jiangsu, China). Mice were housed in a SPF environment with free access to water and food, and maintained on a 12‐h light/12‐h dark cycle. All experimental procedures were approved by the Ethics Committee of Nanjing Drum Tower Hospital, and all procedures were conducted in accordance with the animal guidelines of Nanjing Drum Tower Hospital. Animals were allocated to experimental groups using a simple randomization procedure.

### 4.11 In Vitro and In Vivo Biocompatibility Analysis of AM‐AS@CDs

#### 4.11.1 In Vitro Studies

RTECs, NRK‐52E, and HK‐2 cells were co‐incubated with corresponding CDs at various concentrations (5, 10, 15, 20, 30, 40, 60, 80, and 100 µg/mL) for 24 h. Cell viability was assessed using the Cell Counting Kit‐8 (CCK‐8) assay to evaluate the potential cytotoxicity of AM@CDs, AS@CDs, and AM‐AS@CDs.

#### 4.11.2 In Vivo Studies

Healthy male C57BL/6J mice were treated by intraperitoneal injection of either PBS or 5 mg/kg AM‐AS@CDs. On day 7 post‐injection, blood samples were collected for biochemical analysis, and major organs (including heart, liver, kidneys, spleen, and lungs) were harvested for H&E staining to assess the in vivo biocompatibility of AM‐AS@CDs.

### 4.12 AKI Mouse Model and Therapeutic Experiment

#### 4.12.1 AKI Mouse Model

After at least 7 days of acclimatization, 6–8‐week‐old male C57BL/6J mice were anesthetized via intraperitoneal injection of 2% pentobarbital sodium solution and placed in a supine position on a heated surgical board maintained at 37°C. A 1.5–2 cm midline abdominal incision was made using surgical scissors, and the bilateral renal vessels were carefully isolated under a microscope. Non‐traumatic microvascular clamps were used to occlude both renal pedicles for 25 min, with the kidneys turning from bright red to dark purple, indicating successful ischemia. Reperfusion was confirmed by the return of the kidneys to a bright red color after clamp removal. The abdomen was closed after confirming no bleeding or organ damage, with the kidneys gently repositioned and the incision sutured in layers. In the sham group, renal vessels were exposed but not clamped. Postoperatively, mice were kept warm at room temperature and monitored for survival.

#### 4.12.2 Therapeutic Experiment

After at least 7 days of acclimatization, 6–8‐week‐old male C57BL/6J mice were randomly assigned to five equal groups: Sham, I/R, I/R + AM@CDs, I/R + AS@CDs, and I/R + AM‐AS@CDs. Sham and I/R groups received intraperitoneal vehicle (100 µL PBS), whereas the remaining groups received AM@CDs, AS@CDs, or AM‐AS@CDs (5 mg/kg, 100 µL) at 1 day pre‐I/R, the day of I/R, and 1 day post‐I/R.

### 4.13 Renal Function and Tissue Injury Assessment

Renal function was evaluated by measuring serum CRE and BUN levels using sarcosine oxidase and urease methods, respectively. Renal tissues were fixed in 4% paraformaldehyde, embedded in paraffin, and sectioned at 5 µm thickness for H&E staining. Renal tissue injury was assessed using the Paller pathological scoring system, with scores ranging from 0 to 7, where higher scores indicate more severe damage.

### 4.14 Flow Cytometric Analysis of Immune Cells in Kidneys and Peripheral Blood

Kidneys were harvested, minced into small pieces, and digested with 1 mL of Type I collagenase (1 mg/mL) at 37°C for 30 min on a shaking incubator. The cell suspension was then passed through a 70 µm cell strainer to remove undigested tissue and obtain a single‐cell suspension. Peripheral blood samples were first anticoagulated with EDTA, and 30 µL of anticoagulated blood was incubated with appropriately diluted fluorochrome‐conjugated antibodies for 30 min. Antibodies used in this study included FITC anti‐mouse CD11b, PE anti‐mouse Ly6G, PerCP anti‐mouse CD45, FITC anti‐mouse CD3, APC anti‐mouse F4/80, PE anti‐mouse CD4, and APC anti‐mouse CD8. Flow cytometric data from kidneys and peripheral blood were analyzed using Flowjo software.

### 4.15 WB Analysis

Total protein from renal tissues was lysed in RIPA lysis buffer containing 1% phosphatase inhibitor and 1% protease inhibitor at 4°C. The supernatant was collected and centrifuged. Protein concentration was determined using a BCA protein assay kit. Approximately 20 µg of protein per sample was separated by 10% SDS‐PAGE and transferred to a PVDF membrane. The membrane was blocked with 5% skimmed milk solution or 5% Bovine Serum Albumin (BSA) and incubated with primary antibodies overnight on a shaker at 4°C. Target proteins were detected using an electrochemiluminescence (ECL) system after incubation with secondary antibodies for 1 h at room temperature.

### 4.16 RNA Extraction and qRT‐PCR

Total RNA was extracted from frozen tissues using Trizol reagent. The primers used in this study are listed in Table S1. The expression levels of target genes were normalized to the expression level of β‐Actin using the relative quantification method.

### 4.17 The RNA‐seq Workflow for Kidney Tissue

Mouse kidney tissues were obtained under sterile conditions and immediately flash‐frozen in liquid nitrogen to stabilize RNA. Total RNA was extracted from kidney tissues using a standard method, and RNA integrity was precisely evaluated using an Agilent 2100 Bioanalyzer for strict quality control of RNA samples. mRNA was enriched from total RNA using Oligo (dT) magnetic beads for library construction. The library was quantified using a Qubit fluorometer and quantitative real‐time PCR, and the fragment size distribution was detected using a Bioanalyzer. The libraries were pooled based on the effective concentrations and targeted data amount, and then subjected to Illumina sequencing. After sequencing, raw reads were subjected to quality control using the fastp software, and sequences were aligned to the reference genome using HISAT2 (2.2.1). The number of reads mapped to each gene was calculated using featureCounts (2.0.6), and the FPKM values were calculated based on gene length. Differential expression analysis between two groups was performed using the ‘DESeq2’ R package (1.38.3). GO and KEGG enrichment analyses of DEGs were performed using the ‘clusterProfiler’ R package (4.6.2). GSEA analysis of GO datasets was conducted using the local version of the GSEA analysis tool (available at http://www.broadinstitute.org/gsea/index.jsp). Heatmaps and volcano plots were generated using the ‘ggplot2’ R package (3.5.2).

### 4.18 Statistical Analysis

All statistical analyses were performed using GraphPad Prism 9.5 software. Data were initially tested for normality and homogeneity of variance. Statistical significance between two groups was assessed using Student's *t*‐test. For comparisons involving three or more groups, one‐way analysis of variance (ANOVA) was conducted, followed by Tukey's multiple comparisons test. All data are presented as mean ± standard deviation (SD). Statistical significance was determined based on the following thresholds: ^*^ *p* < 0.05, ^**^ *p* < 0.01, ^***^ *p* < 0.001; ns, not significant.

### 4.19 Ethical Statement

The authors are accountable for all aspects of the work in ensuring that questions related to the accuracy or integrity of any part of the work are appropriately investigated and resolved. The experiments on mice were approved by the Institutional Animal Care and Use Committee, Nanjing University, and all experiments were performed in accordance with relevant guidelines and regulations (Ethical approval number: 2022AE01028).

## Author Contributions

B.X.L. and F.Y.Z. made equal contributions to this research and jointly served as the first authors. X.Z.Z. and L.L. proposed the research idea and oversaw the entire project. B.X.L. designed the experiments, completed most of the experimental work, analyzed the data, and wrote the paper. F.Y.Z. synthesized and characterized the materials. Z.Q.W., X.M.C., C.Z.Y, and Y.P. conducted some of the experiments. J.W.C. performed the experiments and data analysis during the revision period. H.W. and D.Y.F. revised the paper. All authors jointly participated in the writing of the paper and had extensive discussions.

## Funding

The National Natural Science Foundation of China (82570814, 82500836), the Natural Science Foundation of Jiangsu Province (BK20240244), and the Jiangsu Funding Program for Excellent Postdoctoral Talent (2022ZB683).

## Conflicts of Interest

The authors declare no conflicts of interest.

## Supporting information




**Supporting File**: advs75596‐sup‐0001‐SuppMat.docx.

## Data Availability

The data that support the findings of this study are available from the corresponding author upon reasonable request.

## References

[1] N. C. Chesnaye , J. J. Carrero , M. Hecking , and K. J. Jager , “Differences In The Epidemiology, Management And Outcomes Of Kidney Disease In Men And Women,” Nature Reviews Nephrology 20, no. 1 (2024): 7–20, 10.1038/s41581-023-00784-z. 37985869

[2] M. Ostermann , N. Lumlertgul , R. Jeong , E. See , M. Joannidis , and M. James , “Acute Kidney Injury,” The Lancet 405, no. 10474 (2025): 241–256, 10.1016/S0140-6736(24)02385-7. 39826969

[3] J. Yan , W. Yan , and D. Zhang , “Mmu_circ_0005698/hsa_circ_0085381/miR‐532‐3p/Arhgdib Axis Mediates The Ischemic Progression Of Acute Kidney Injury,” International Immunopharmacology 163 (2025): 115207, 10.1016/j.intimp.2025.115207. 40682984

[4] B. Kolbrink , F. A. von Samson‐Himmelstjerna , J. M. Murphy , and S. Krautwald , “Role Of Necroptosis In Kidney Health And Disease,” Nature Reviews Nephrology 19, no. 5 (2023): 300–314, 10.1038/s41581-022-00658-w. 36596919

[5] I. H. Schulman , K. Chan , J. S. Der , et al., “Readmission and Mortality After Hospitalization With Acute Kidney Injury,” American Journal of Kidney Diseases 82, no. 1 (2023): 63–74, 10.1053/j.ajkd.2022.12.008. 37115159 PMC10293057

[6] L. He , Q. Wei , J. Liu , et al., “AKI on CKD: Heightened Injury, Suppressed Repair, And The Underlying Mechanisms,” Kidney International 92, no. 5 (2017): 1071–1083, 10.1016/j.kint.2017.06.030. 28890325 PMC5683166

[7] J. Himmelfarb , E. McMonagle , S. Freedman , et al., “Oxidative Stress Is Increased In Critically Ill Patients With Acute Renal Failure,” Journal of the American Society of Nephrology 15, no. 9 (2004): 2449–2456, 10.1097/01.Asn.0000138232.68452.3b. 15339994

[8] H. K. Eltzschig and T. Eckle , “Ischemia And Reperfusion—From Mechanism To Translation,” Nature Medicine 17, no. 11 (2011): 1391–1401, 10.1038/nm.2507. PMC3886192 22064429

[9] G. Y. Chen and G. Nuñez , “Sterile Inflammation: Sensing And Reacting To Damage,” Nature Reviews Immunology 10, no. 12 (2010): 826–837, 10.1038/nri2873. PMC3114424 21088683

[10] J. Liu , X. Sun , J. Liang , and S. Song , “Eugenol Alleviates Renal Ischemia‐Reperfusion Injury Induced‐Endoplasmic Reticulum Stress Via Activating Sestrin2,” Clinics 80 (2025): 100627, 10.1016/j.clinsp.2025.100627. 40138864 PMC11985136

[11] X. Wang , S. Wu , Y. Jiang , et al., “Anwulignan Alleviates IRI By The Activation of Nrf2/HO‐1 Signaling Pathway And Inhibiting NLRP3‐Caspase‐1‐GSDMD‐Mediated Pyroptosis In Rats,” Tissue and Cell 93 (2025): 102775, 10.1016/j.tice.2025.102775. 39923645

[12] P. Bhargava and R. G. Schnellmann , “Mitochondrial Energetics In The Kidney,” Nature Reviews Nephrology 13, no. 10 (2017): 629–646, 10.1038/nrneph.2017.107. 28804120 PMC5965678

[13] H. Kocaturk , F. Bedir , M. S. Altay , et al., “The Effect Of Desloratadine On Ischemia Reperfusion Induced Oxidative And Inflammatory Renal Injury In Rats,” Renal Failure 42, no. 1 (2020): 531–538, 10.1080/0886022x.2020.1769656. 32524906 PMC7946030

[14] B. B. Ratliff , M. M. Rabadi , R. Vasko , K. Yasuda , and M. S. Goligorsky , “Messengers Without Borders,” Journal of the American Society of Nephrology 24, no. 4 (2013): 529–536, 10.1681/asn.2012060633. 23349311

[15] C. Kurts , U. Panzer , H. J. Anders , and A. J. Rees , “The Immune System And Kidney Disease: Basic Concepts And Clinical Implications,” Nature Reviews Immunology 13, no. 10 (2013): 738–753, 10.1038/nri3523. 24037418

[16] Y. Fu , W. Wang , N. Gong , et al., “Neutrophil And Neutrophil Extracellular Traps In Acute Kidney Injury: From Mechanisms To Treatments,” Frontiers in Immunology 16 (2025): 1688207, 10.3389/fimmu.2025.1688207. 41169399 PMC12568571

[17] D. Hammouri , T. Weis , and L. J. Siskind , “Macrophage Plasticity and Functional Dynamics in Acute Kidney Injury and Its Progression to Chronic Kidney Disease,” Seminars in Nephrology (2025): 151672, 10.1016/j.semnephrol.2025.151672. 41193294 PMC12608096

[18] K. Lee , H. R. Jang , and H. Rabb , “Lymphocytes And Innate Immune Cells In Acute Kidney Injury And Repair,” Nature Reviews Nephrology 20, no. 12 (2024): 789–805, 10.1038/s41581-024-00875-5. 39095505

[19] C. Zhang , Z. Xiang , P. Yang , L. Zhang , J. Deng , and X. Liao , “Advances in Nano‐Immunomodulatory Systems for the Treatment of Acute Kidney Injury,” Advanced Science 12, no. 17 (2025): 2409190, 10.1002/advs.202409190. 40145715 PMC12061249

[20] P. Liu , X. Li , W. Lv , and Z. Xu , “Inhibition of CXCL1‐CXCR2 Axis Ameliorates Cisplatin‐Induced Acute Kidney Injury By Mediating Inflammatory Response,” Biomedicine & Pharmacotherapy 122 (2020): 109693, 10.1016/j.biopha.2019.109693. 31812015

[21] J. Wu , X. Wang , Q. Wang , et al., “Nanomaterials With Enzyme‐Like Characteristics (Nanozymes): Next‐Generation Artificial Enzymes (II),” Chemical Society Reviews 48, no. 4 (2019): 1004–1076, 10.1039/c8cs00457a. 30534770

[22] D. Chao , Q. Dong , Z. Yu , et al., “Specific Nanodrug for Diabetic Chronic Wounds Based on Antioxidase‐Mimicking MOF‐818 Nanozymes,” Journal of the American Chemical Society 144, no. 51 (2022): 23438–23447, 10.1021/jacs.2c09663. 36512736

[23] X. Zhang , X. Chen , and Y. Zhao , “Nanozymes: Versatile Platforms for Cancer Diagnosis and Therapy,” Nanomicro Letter 14, no. 1 (2022): 95, 10.1007/s40820-022-00828-2. PMC8986955 35384520

[24] X. Y. Du , C. F. Wang , G. Wu , and S. Chen , “The Rapid and Large‐Scale Production of Carbon Quantum Dots and their Integration With Polymers,” Angewandte Chemie International Edition 60, no. 16 (2021): 8585–8595, 10.1002/anie.202004109. 32410267

[25] L. Yao , M.‐M. Zhao , Q.‐W. Luo , et al., “Carbon Quantum Dots‐Based Nanozyme From Coffee Induces Cancer Cell Ferroptosis to Activate Antitumor Immunity,” ACS Nano 16, no. 6 (2022): 9228–9239, 10.1021/acsnano.2c01619. 35622408

[26] A. Xu , G. Wang , Y. Li , et al., “Carbon‐Based Quantum Dots With Solid‐State Photoluminescent: Mechanism, Implementation, and Application,” Small 16, no. 48 (2020): 2004621, 10.1002/smll.202004621. 33145929

[27] F. Gao , J. Huang , Y. Ruan , et al., “Unraveling the Structure Transition and Peroxidase Mimic Activity of Copper Sites Over Atomically Dispersed Copper‐Doped Carbonized Polymer Dots,” Angewandte Chemie 62, no. 7 (2023): 202214042, 10.1002/anie.202214042. 36565238

[28] H. Sun , Y. Zhou , J. Ren , and X. Qu , “Carbon Nanozymes: Enzymatic Properties, Catalytic Mechanism, and Applications,” Angewandte Chemie International Edition 57, no. 30 (2018): 9224–9237, 10.1002/anie.201712469. 29504678

[29] B. Kong , T. Yang , F. Cheng , et al., “Carbon Dots As Nanocatalytic Medicine For Anti‐Inflammation Therapy,” Journal of Colloid and Interface Science 611 (2022): 545–553, 10.1016/j.jcis.2021.12.107. 34971965

[30] Y. Zhang , W. Gao , Y. Ma , et al., “Integrating Pt Nanoparticles With Carbon Nanodots To Achieve Robust Cascade Superoxide Dismutase‐Catalase Nanozyme For Antioxidant Therapy,” Nano Today 49 (2023): 101768, 10.1016/j.nantod.2023.101768.

[31] Y. Li , W. Li , X. Yang , et al., “Salvia Miltiorrhiza‐Derived Carbon Dots as Scavengers of Reactive Oxygen Species for Reducing Oxidative Damage of Plants,” ACS Applied Nano Materials 4, no. 1 (2021): 113–120, 10.1021/acsanm.0c02419.

[32] X. Zhou , J. Zhou , J. Ren , Z. Qu , and T. Zhang , “Progress in the Study of Extraction Methods and Pharmacological Effects of Traditional Chinese Medicine‐Derived Carbon Dots,” Molecules 30, no. 19 (2025): 4015, 10.3390/molecules30194015. 41097434 PMC12526297

[33] W.‐J. Liu , Y.‐Z. Ma , J.‐X. Li , et al., “Structural Characterization Of A Polysaccharide From Qi‐Gui Herb Pair And Its Anti‐Tumor Activity In Colon Cancer Cells,” Frontiers in Pharmacology 16 (2025): 1557151, 10.3389/fphar.2025.1557151. 40196375 PMC11973367

[34] Y. Guo , P. Yang , Z. Wu , S. Zhang , and F. You , “Mechanisms of Astragalus membranaceus (Fisch.) Bge. var. mongholicus (Bge.) Hsiao (huang qi) and Angelica sinensis (Oliv.) Diels (dang gui) in Ameliorating Hypoxia and Angiogenesis to Delay Pulmonary Nodule Malignant Transformation,” Integrative Cancer Therapies 24 (2025): 15347354241311917, 10.1177/15347354241311917. 39882753 PMC11780663

[35] L. Liu , R. Wang , W. Gao , et al., “Drug Pairs Of Huangqi And Dnggui Alleviates Pyroptosis By Promoting Autophagy Activity Via AMPK/mTOR Signaling Pathway In Middle‐Cerebral Artery Occlusion/Reperfusion In Rats,” Journal of Ethnopharmacology 337, no. 3 (2025): 118982, 10.1016/j.jep.2024.118982. 39454707

[36] M. Dong , J. Li , D. Yang , M. Li , and J. Wei , “Biosynthesis and Pharmacological Activities of Flavonoids, Triterpene Saponins and Polysaccharides Derived From Astragalus membranaceus,” Molecules 28, no. 13 (2023): 5018, 10.3390/molecules28135018. 37446680 PMC10343288

[37] X. Zhi , C. Ren , Q. Li , et al., “Therapeutic Potential Of Angelica Sinensis In Addressing Organ Fibrosis: A Comprehensive Review,” Biomedicine & Pharmacotherapy 173 (2024): 116429, 10.1016/j.biopha.2024.116429. 38490157

[38] J. Zhang , L. Zou , Q. Li , et al., “Carbon Dots Derived From Traditional Chinese Medicines With Bioactivities: A Rising Star in Clinical Treatment,” ACS Applied Bio Materials 6, no. 10 (2023): 3984–4001, 10.1021/acsabm.3c00462. 37707491

[39] W.‐K. Luo , L.‐L. Zhang , Z.‐Y. Yang , et al., “Herbal Medicine Derived Carbon Dots: Synthesis And Applications In Therapeutics, Bioimaging And Sensing,” Journal of Nanobiotechnology 19, no. 1 (2021): 320, 10.1186/s12951-021-01072-3. 34645456 PMC8513293

[40] H. Huang , Z. Shen , B. Chen , et al., “Selenium‐Doped Two‐Photon Fluorescent Carbon Nanodots For In‐Situ Free Radical Scavenging In Mitochondria,” Journal of Colloid and Interface Science 567 (2020): 402–409, 10.1016/j.jcis.2020.02.011. 32078945

[41] J. Shi , T. Yin , and W. Shen , “Effect Of Surface Modification On The Peroxidase‐Like Behaviors Of Carbon Dots,” Colloids and Surfaces B: Biointerfaces 178 (2019): 163–169, 10.1016/j.colsurfb.2019.03.012. 30856585

[42] W. Gao , J. He , L. Chen , et al., “Deciphering The Catalytic Mechanism Of Superoxide Dismutase Activity Of Carbon Dot Nanozyme,” Nature Communications 14, no. 1 (2023): 160, 10.1038/s41467-023-35828-2. PMC9834297 36631476

[43] C. Kang , S. Tao , F. Yang , and B. Yang , “Aggregation And Luminescence In Carbonized Polymer Dots,” Aggregate 3, no. 2 (2022): 169, 10.1002/agt2.169.

[44] Y. Deng , Z. Liu , X. Zhu , Y. Wang , X. Feng , and J. Yang , “Kidney‐Targeted Nanoplatforms: Strategies And Applications,” Theranostics 16, no. 6 (2026): 3011–3031, 10.7150/thno.126217. 41510170 PMC12775796

[45] A. Truskewycz , H. Yin , N. Halberg , et al., “Carbon Dot Therapeutic Platforms: Administration, Distribution, Metabolism, Excretion, Toxicity, and Therapeutic Potential,” Small 18, no. 16 (2022): 2106342, 10.1002/smll.202106342. 35088534

[46] L. Wang , Y. Wang , T. Xu , et al., “Gram‐Scale Synthesis Of Single‐Crystalline Graphene Quantum Dots With Superior Optical Properties,” Nature Communications 5 (2014): 5357, 10.1038/ncomms6357. 25348348

[47] S. H. Yang , S. K. Park , and Y. C. Kang , “MOF‐Derived CoSe_2_@N‐Doped Carbon Matrix Confined in Hollow Mesoporous Carbon Nanospheres as High‐Performance Anodes for Potassium‐Ion Batteries,” Nanomicro Letter 13, no. 1 (2020): 9, 10.1007/s40820-020-00539-6. PMC8187686 34138196

[48] L. Xia , Q. Yang , K. Fu , et al., “Unveiling The Renoprotective Mechanisms Of Self‐Assembled Herbal Nanoparticles From Scutellaria Barbata And Scleromitrion Diffusum In Acute Kidney Injury: A Nano‐TCM Approach,” Acta Pharmaceutica Sinica B 15, no. 8 (2025): 4265–4284, 10.1016/j.apsb.2025.05.024. 40893687 PMC12399204

[49] K. Dehvari , S. H. Chiu , J. S. Lin , W. M. Girma , Y. C. Ling , and J. Y. Chang , “Heteroatom Doped Carbon Dots With Nanoenzyme Like Properties As Theranostic Platforms For Free Radical Scavenging, Imaging, And Chemotherapy,” Acta Biomaterialia 114 (2020): 343–357, 10.1016/j.actbio.2020.07.022. 32682058

[50] K. Chotinaruemol , P. Leurcharusmee , S. C. Chattipakorn , N. Chattipakorn , and N. Apaijai , “Dexmedetomidine Mitigation Of Renal Ischaemia–Reperfusion Injury: Comprehensive Insights From Cellular Mechanisms To Clinical Application,” British Journal of Anaesthesia 134, no. 5 (2025): 1350–1372, 10.1016/j.bja.2025.02.006. 40082177

[51] K. Tejchman , K. Kotfis , and J. Sieńko , “Biomarkers and Mechanisms of Oxidative Stress—Last 20 Years of Research With an Emphasis on Kidney Damage and Renal Transplantation,” International Journal of Molecular Sciences 22, no. 15 (2021): 8010, 10.3390/ijms22158010. 34360776 PMC8347360

[52] A. Salvati , C. Åberg , T. dos Santos , et al., “Experimental And Theoretical Comparison Of Intracellular Import Of Polymeric Nanoparticles And Small Molecules: Toward Models Of Uptake Kinetics,” Nanomedicine: Nanotechnology, Biology and Medicine 7, no. 6 (2011): 818–826, 10.1016/j.nano.2011.03.005. 21453790

[53] T. Thomsen , A. B. Ayoub , D. Psaltis , and H. A. Klok , “Fluorescence‐Based and Fluorescent Label‐Free Characterization of Polymer Nanoparticle Decorated T Cells,” Biomacromolecules 22, no. 1 (2021): 190–200, 10.1021/acs.biomac.0c00969. 32869972

[54] D. Troise , B. Infante , S. Mercuri , B. Lindholm , K. Kublickiene , and G. Stallone , “Exploring the Immunological Landscape of Ischemia/Reperfusion Injury and Graft Rejection in Kidney Transplantation: Shared Mechanisms and Insights,” Cells 14, no. 18 (2025): 1443, 10.3390/cells14181443. 41002408 PMC12468464

[55] I. Cobo , J. Murillo‐Saich , M. Alishala , et al., “Particle Uptake By Macrophages Triggers Bifurcated Transcriptional Pathways That Differentially Regulate Inflammation And Lysosomal Gene Expression,” Immunity 58, no. 4 (2025): 826–842, 10.1016/j.immuni.2025.02.023. 40118070 PMC12093573

[56] L. Zhang , M. Yu , J. Deng , et al., “Chemokine Signaling Pathway Involved in CCL2 Expression in Patients With Rheumatoid Arthritis,” Yonsei Medical Journal 56, no. 4 (2015): 1134–1142, 10.3349/ymj.2015.56.4.1134. 26069140 PMC4479845

