# Supplementary material for: Engineered Carbon Dots from a Traditional Herb Pair Orchestrate Concurrent Antioxidant and AP‐1‐Mediated Inflammation to Attenuate Renal Ischemia‐Reperfusion Injury
Source: Adv Sci (Weinh). 2026 May 12;13(44):e75596. doi: 10.1002/advs.75596 (PMC13336125; doi:10.1002/advs.75596)
Supplement: Supplementary file 1 [file ADVS-13-e75596-s001.docx]

Supporting Information

**Engineered Carbon Dots from a Traditional Herb Pair Orchestrate Concurrent Antioxidant and AP-1-Mediated Inflammation to Attenuate Renal Ischemia-Reperfusion Injury**

*Bixiao Liu^1#^, Fuying Zhu^1,2#^, Zhuqing Wang^1^, Jiawen Chen^2^, Xiaomiao Cui^2^, Congzhong Yang^2^, Yao Peng^1^, Dengyuan Feng^1*^, Li Lu^1*^, Hui Wei^1,2*^, and Xiaozhi Zhao^1*^*

**Figure S1. Schematic of the hydrothermal synthesis process.**

**Figure S2. Photograph of the CDs solutions under 365 nm UV light (1: AM-AS@CDs, 2: AM@CDs, 3: AS@CDs, 4: water).**

**Figure S3. Quantitative analysis of the carbon (C), nitrogen (N), and oxygen (O) content in AM@CDs, AS@CDs, and AM-AS@CDs.**

**Figure S4.** **High-resolution O 1*s* XPS spectra of (a) AM@CDs, (b) AS@CDs, and (c) AM-AS@CDs.**

**Figure S5. High-resolution N 1*s* XPS spectra of (a) AM@CDs, (b) AS@CDs, and (c) AM-AS@CDs.**

**Figure S6. Dose-response curves of AM@CDs, AS@CDs, and AM-AS@CDs for superoxide anion scavenging.** The percentage of superoxide anion scavenging was measured at multiple concentrations of each carbon dot sample. The specific SOD-like activities (U/mg) were calculated from these curves. Error bars represent standard deviations from three independent measurements.

**Figure S7. Assessment the CAT-like activity of AM-AS@CDs, AM@CDs, and AS@CDs.**

**Figure S8. Assessment the POD-like activity of AM-AS@CDs, AM@CDs, and AS@CDs.**

**Figure S9. Assessment the OXD-like activity of AM-AS@CDs, AM@CDs, and AS@CDs.**

**Figure S10. The in vitro safety of AM-AS@CDs with different precursor mass ratios.** (a-i) Relative viability of NRK-52E, HK-2 and PTECs after 24 h exposure to graded concentrations of AM-AS@CDs, AM@CDs or AS@CDs (n = 5). Data are presented as mean ± SD from at least five independent experiments. Statistical comparisons were performed using one-way ANOVA and t-test; ns, not significant.

**Figure S11. In vitro antioxidant and anti-apoptotic activities of AM-AS@CDs with different precursor mass ratios.** (a) Flow cytometric analysis of ROS levels in NRK-52E cells. (b) Quantification of relative ROS levels normalized to the control group (n = 3). (c) Representative flow cytometry plots and quantitative analysis of apoptosis in NRK-52E cells (n = 3). Cells in the Q2 (Annexin V^+^ PI^+^) and Q3 (Annexin V^+^ PI^-^) quadrants were defined as late and early apoptotic cells respectively. Cells in these two quadrants were considered apoptotic. “+” denotes H_2_O_2_-stimulated injury model, “-” denotes untreated control. MFI, mean fluorescence intensity. Data are presented as mean ± SD from at least three independent experiments. Statistical comparisons were performed using one-way ANOVA and t-test; *p < 0.05, ***p < 0.001.

**Figure S12.** **Assessment of SOD-like activity for surface-modified AM-AS@CDs.**

**Figure S13. In vitro antioxidant and anti-apoptotic activities of AM-AS@CDs at different concentrations.** (a) Flow cytometric analysis of ROS levels in NRK-52E cells. (b) Quantification of relative ROS levels normalized to the control group (n = 3). (c, d) Representative flow cytometry plots and quantitative analysis of apoptosis in NRK-52E cells (n = 3). Cells in the Q2 (Annexin V^+^ PI^+^) and Q3 (Annexin V^+^ PI^-^) quadrants were defined as late and early apoptotic cells respectively. Cells in these two quadrants were considered apoptotic. “+” denotes H_2_O_2_-stimulated injury model, “-” denotes untreated control. MFI, mean fluorescence intensity. Data are presented as mean ± SD from at least three independent experiments. Statistical comparisons were performed using one-way ANOVA and t-test; ***p < 0.001.

**Figure S14. In vitro antioxidant and anti-apoptotic activities of AM-AS@CDs and NAC.** (a) Flow cytometric analysis of ROS levels in NRK-52E cells. (b) Quantification of relative ROS levels normalized to the control group (n = 3). (c, d) Representative flow cytometry plots and quantitative analysis of apoptosis in NRK-52E cells (n = 3). Cells in the Q2 (Annexin V^+^ PI^+^) and Q3 (Annexin V^+^ PI^-^) quadrants were defined as late and early apoptotic cells respectively. Cells in these two quadrants were considered apoptotic. “+” denotes H_2_O_2_-stimulated injury model, “-” denotes untreated control. MFI, mean fluorescence intensity. Data are presented as mean ± SD from at least three independent experiments. Statistical comparisons were performed using one-way ANOVA and t-test; *p < 0.05, **p < 0.01, ***p < 0.001.

**Figure S15. Antioxidant and anti-apoptotic activities of AM-AS@CDs in HK-2 cells.** (a) Fluorescence micrographs of intracellular ROS in HK-2 cells under indicated treatments (scale bar = 50 μm). (b) Flow cytometric analysis of ROS levels in HK-2 cells. (c) Quantification of relative ROS levels normalized to the control group (n = 3). (d, e) Representative flow cytometry plots and quantitative analysis of apoptosis in HK-2 cells (n = 3). Cells in the Q2 (Annexin V^+^ PI^+^) and Q3 (Annexin V^+^ PI^-^) quadrants were defined as late and early apoptotic cells respectively. Cells in these two quadrants were considered apoptotic. “+” denotes H_2_O_2_-stimulated injury model, “-” denotes untreated control. MFI, mean fluorescence intensity. Data are presented as mean ± SD from at least three independent experiments. Statistical comparisons were performed using one-way ANOVA and t-test; *p < 0.05, ***p < 0.001; ns, not significant.

**Figure S16. Antioxidant and anti-apoptotic activities of AM-AS@CDs in RTECs.** (a) Fluorescence micrographs of intracellular ROS in RTECs under indicated treatments (scale bar = 50 μm). (b) Flow cytometric analysis of ROS levels in RTECs. (c) Quantification of relative ROS levels normalized to the control group (n = 3). (d, e) Representative flow cytometry plots and quantitative analysis of apoptosis in RTECs (n = 3). Cells in the Q2 (Annexin V^+^ PI^+^) and Q3 (Annexin V^+^ PI^-^) quadrants were defined as late and early apoptotic cells respectively. Cells in these two quadrants were considered apoptotic. Cells in these two quadrants were considered apoptotic. “+” denotes H_2_O_2_-stimulated injury model, “-” denotes untreated control. MFI, mean fluorescence intensity. Data are presented as mean ± SD from at least three independent experiments. Statistical comparisons were performed using one-way ANOVA and t-test; *p < 0.05, **p < 0.01, ***p < 0.001; ns, not significant.

**Figure S17. Renoprotective efficacy of AM-AS@CDs** **under two dosing regimens after I/R.** (a) Schematic timeline illustrating the prophylactic and therapeutic regimen for different treatment groups. (b, c) Functional assessment of kidney injury 48 h post-reperfusion (n = 4). CRE, creatinine; BUN, blood urea nitrogen. (d, e) Renal mRNA expression levels of *Kim-1* and *Lcn2* in each group (n = 4). (f) Representative H&E-stained histological images (scale bar = 50 µm). (g) Damage scores of kidney tissues (n = 3). Pro-thera, prophylactic-therapeutic regimen; Post, post-reperfusion regimen. Data are presented as mean ± SD from at least three independent experiments. Statistical comparisons were performed using one-way ANOVA and t-test; *p < 0.05, ***p < 0.001.

**Figure S18. Biosafety analysis of AM-AS@CDs.** (a) Representative H&E-stained histological images (scale bar = 50 µm). (b) Body weight of mice in different treatment groups (n = 3). (c) The ratio of bilateral kidney weight/body weight (n = 3). (d, e) Functional assessment of kidney (n = 3). CRE, creatinine; BUN, blood urea nitrogen. (f, g) Functional assessment of liver (n = 3). ALT, alanine aminotransferase; AST, aspartate aminotransferase. Data are presented as mean ± SD from at least three independent experiments. Statistical comparisons were performed using one-way ANOVA and t-test; ns, not significant.

**Figure S19. Representative flow cytometric plots and quantitative analysis of circulating CD4^+^/CD8^+^ T lymphocytes (n = 5).** Data are presented as mean ± SD from at least five independent experiments. Statistical comparisons were performed using one-way ANOVA and t-test; ns, not significant.

**Figure S20. Change in inflammatory levels after I/R treatment.** (a, b) WB analysis of NF-κB expression in each group. (c, d) The results of qRT-PCR of *TNF-α* (n = 5) and *IL-1β* (n = 3) in different treatment groups. Data are presented as mean ± SD from at least three independent experiments. Statistical comparisons were performed using one-way ANOVA and t-test; *p < 0.05, **p < 0.01, ***p < 0.001.

**Figure S21. Pearson correlation analysis between samples.**

**Figure S22. PCA analysis of the Sham, I/R, and I/R + AM-AS@CDs.**

**Figure S23. The results of qRT-PCR of *CAT* (a) and *GPX4* (b) in different treatment groups (n = 4).** Data are presented as mean ± SD from at least four independent experiments. Statistical comparisons were performed using one-way ANOVA and t-test; *p < 0.05, ***p < 0.001.

**Table S1. Primers used in this study**

| Gene | Primer Name | Sequence (5'-3') |
| --- | --- | --- |
| Lcn2 | Lcn2-Fwd | TGGCCCTGAGTGTCATGTG |
| Lcn2 | Lcn2-Rev | CTCTTGTAGCTCATAGATGGTGC |
| Kim-1 | Kim-1-Fwd | ACATATCGTGGAATCACAACGAC |
| Kim-1 | Kim-1- Rev | ACAAGCAGAAGATGGGCATTG |
| Ccl2 | Ccl2-Fwd | TTAAAAACCTGGATCGGAACCAA |
| Ccl2 | Ccl2-Rev | GCATTAGCTTCAGATTTACGGGT |
| Ccl7 | Ccl7-Fwd | GCTGCTTTCAGCATCCAAGTG |
| Ccl7 | Ccl7-Rev | CCAGGGACACCGACTACTG |
| Ccl20 | Ccl20-Fwd | GCCTCTCGTACATACAGACGC |
| Ccl20 | Ccl20-Rev | CCAGTTCTGCTTTGGATCAGC |
| Cxcl1 | Cxcl1-Fwd | CTGGGATTCACCTCAAGAACATC |
| Cxcl1 | Cxcl1-Rev | CAGGGTCAAGGCAAGCCTC |
| TNF-α | TNF-α-Fwd | CCCTCACACTCAGATCATCTTCT |
| TNF-α | TNF-α-Rev | GCTACGACGTGGGCTACAG |
| IL-1β | IL-1β-Fwd | GCAACTGTTCCTGAACTCAACT |
| IL-1β | IL-1β-Rev | ATCTTTTGGGGTCCGTCAACT |
| SOD1 | SOD1-Fwd | AACCAGTTGTGTTGTCAGGAC |
| SOD1 | SOD1-Rev | CCACCATGTTTCTTAGAGTGAGG |
| SOD2 | SOD2-Fwd | CAGACCTGCCTTACGACTATGG |
| SOD2 | SOD2-Rev | CTCGGTGGCGTTGAGATTGTT |
| SOD3 | SOD3-Fwd | CCTTCTTGTTCTACGGCTTGC |
| SOD3 | SOD3-Rev | TCGCCTATCTTCTCAACCAGG |
| GPX4 | GPX4-Fwd | GATGGAGCCCATTCCTGAACC |
| GPX4 | GPX4-Rev | CCCTGTACTTATCCAGGCAGA |
| CAT | CAT-Fwd | AGCGACCAGATGAAGCAGTG |
| CAT | CAT-Rev | TCCGCTCTCTGTCAAAGTGTG |
| Fosl1 | Fosl1-Fwd | ATGTACCGAGACTACGGGGAA |
| Fosl1 | Fosl1-Rev | CTGCTGCTGTCGATGCTTG |
| c-Jun | c-Jun-Fwd | CCTTCTACGACGATGCCCTC |
| c-Jun | c-Jun-Rev | GGTTCAAGGTCATGCTCTGTTT |
| S100a8 | S100a8-Fwd | AAATCACCATGCCCTCTACAAG |
| S100a8 | S100a8-Rev | CCCACTTTTATCACCATCGCAA |
| S100a9 | S100a9-Fwd | ATACTCTAGGAAGGAAGGACACC |
| S100a9 | S100a9-Rev | TCCATGATGTCATTTATGAGGGC |
| β-Actin | β-Actin-Fwd | GGCTGTATTCCCCTCCATCG |
| β-Actin | β-Actin-Rev | CCAGTTGGTAACAATGCCATGT |
